# Effects of Arabinoxylan and Resistant Starch on Intestinal Microbiota and Short-Chain Fatty Acids in Subjects with Metabolic Syndrome: A Randomised Crossover Study

**DOI:** 10.1371/journal.pone.0159223

**Published:** 2016-07-19

**Authors:** Stine Hald, Anne Grethe Schioldan, Mary E. Moore, Anders Dige, Helle Nygaard Lærke, Jørgen Agnholt, Knud Erik Bach Knudsen, Kjeld Hermansen, Maria L. Marco, Søren Gregersen, Jens F. Dahlerup

**Affiliations:** 1 Department of Hepatology and Gastroenterology, Aarhus University Hospital, Aarhus, Denmark; 2 Department of Endocrinology and Internal Medicine, Aarhus University Hospital, Aarhus, Denmark; 3 Department of Food Science and Technology, University of California Davis, Davis, California, United States of America; 4 Department of Animal Science, Aarhus University, Tjele, Denmark; Max Rubner-Institut, GERMANY

## Abstract

Recently, the intestinal microbiota has been emphasised as an important contributor to the development of metabolic syndrome. Dietary fibre may exert beneficial effects through modulation of the intestinal microbiota and metabolic end products. We investigated the effects of a diet enriched with two different dietary fibres, arabinoxylan and resistant starch type 2, on the gut microbiome and faecal short-chain fatty acids. Nineteen adults with metabolic syndrome completed this randomised crossover study with two 4-week interventions of a diet enriched with arabinoxylan and resistant starch and a low-fibre Western-style diet. Faecal samples were collected before and at the end of the interventions for fermentative end-product analysis and 16S ribosomal RNA bacterial gene amplification for identification of bacterial taxa. Faecal carbohydrate residues were used to verify compliance. The diet enriched with arabinoxylan and resistant starch resulted in significant reductions in the total species diversity of the faecal-associated intestinal microbiota but also increased the heterogeneity of bacterial communities both between and within subjects. The proportion of *Bifidobacterium* was increased by arabinoxylan and resistant starch consumption (*P*<0.001), whereas the proportions of certain bacterial genera associated with dysbiotic intestinal communities were reduced. Furthermore, the total short-chain fatty acids (*P*<0.01), acetate (*P*<0.01) and butyrate concentrations (*P*<0.01) were higher by the end of the diet enriched with arabinoxylan and resistant starch compared with those resulting from the Western-style diet. The concentrations of isobutyrate (*P* = 0.05) and isovalerate (*P* = 0.03) decreased in response to the arabinoxylan and resistant starch enriched diet, indicating reduced protein fermentation. In conclusion, arabinoxylan and resistant starch intake changes the microbiome and short-chain fatty acid compositions, with potential beneficial effects on colonic health and metabolic syndrome.

**Trial Registration:**

ClinicalTrials.gov NCT01618526

## Introduction

Increased consumption of dietary fibre (DF) has been shown to improve components of metabolic syndrome (MetS), including dyslipidaemia, insulin sensitivity and abdominal obesity,[1–3] which are well-known risk factors for cardiovascular disease and type 2 diabetes. These beneficial effects are most likely modulated through the actions of DF on certain bacteria in the colon[4] and the capabilities of these bacteria to produce an array of small organic molecules, including short-chain fatty acids (SCFAs).[5] Increased production of SCFAs in general and of butyrate in particular may protect against diet-induced obesity and improve insulin sensitivity.[6–8] SCFA production depends on the type of ingested DF, the microbiota, the colonic transit time and the colonic pH.[9]

Resistant starch (RS) is a homo-polysaccharide of glucose[10] resistant to digestion in the upper gastro-intestinal tract[11] because of physical indigestibility (type 1), granularity (type 2), retrogradation during heating (type 3), or chemical modification (type 4).[10] RS type 2 is a readily available pure form of RS that can be obtained in sufficient quantities for human studies. Previously, RS type 2 has been shown to augment butyrate production[12, 13] and to modify the composition of the intestinal microbiota[5, 14–16] typically by increasing the proportions of *Bifidobacterium species*, *Ruminococcus bromii*[14, 17] and *Eubacterium rectale*.[14] Studies on RS type 3 have also been conducted and shown enhancement of *Ruminococcus bromii* and *Eubacterium rectale*,[18, 19] whereas RS type 4 apparently demonstrate different functional effects on the microbiota by augmentation of *Bifidobacterium adolescentis* and *Parabacteroides distasonis*.[14]

Arabinoxylan (AX) is a hetero-polysaccharide consisting of a linear xylose backbone with variable arabinose substitution and cross-linking with ferulic acid, depending on the grain species and fractions.[20] It is the main DF component in wheat and rye[20] and has also been shown to enhance SCFA and butyrate production.[3, 21] The proportions of *Bacteroides*, *Prevotella*, *Roseburia* and *Bifidobacterium* species have been shown to be increased after an AX-enriched high-fat diet in mice,[22] whereas AX provided in a whole-grain rye diet in a human intervention study failed to show changes in the microbiota.[23] Both RS and AX have been found to improve metabolic parameters in humans,[24–26] but there is little knowledge of how these fibres in combination influence the microbiome and SCFA production in prediabetic subjects, i.e. those with MetS.

The objectives of the current crossover study of subjects with MetS were to determine how an AX- and RS-enriched diet, hereafter referred to as a healthy-carbohydrate diet (HCD), alters the microbiome and SCFA concentrations in faeces compared with a low-fibre Western-style diet (WSD). We hypothesised that AX and RS would beneficially change the intestinal microbiota and enhance colonic SCFA production in general and butyrate in particular compared with the WSD.

## Materials and Methods

### Study design and subjects

The dietary intervention study was conducted according to the guidelines in the Declaration of Helsinki, and all procedures involving human subjects were approved by the Central Denmark Region Committees on Biomedical Research Ethics (journal no. 1-10-72-122-12) March 29 2012 (S1 Information). Written informed consent was obtained from all subjects prior to screening. The study was registered at ClinicalTrials.gov ID: NCT01618526 (May 30 2012) and NCT01584427 (April 20 2012). The delay between approval and registration was due to maternity leave for one of the study coordinators. We confirm that all ongoing and related trials for this intervention are registered.

The study was performed as a randomised crossover, open-label study with two 4-week intervention periods and an intermediate washout period of a minimum of four weeks. Recruitment took place via advertisements in local newspapers from May 9 2012 to November 30 2012. The study was carried out at Aarhus University Hospital, Aarhus, Denmark between June 2 2012 and April 11 2013. Twenty-two subjects between 39 and 75 years of age with MetS[27] were included in the study. Two more subjects were included than original described in the trial protocol. We excluded subjects if they had a history of diabetes or gastrointestinal disease or hepatic, renal, cardiovascular or uncontrolled metabolic disease. Alcohol abuse, the daily use of non-steroidal anti-inflammatory drugs, pregnancy and lactation were additional exclusion criteria.

### Intervention diets

The HCD was formulated with a high concentration of DF based on AX- and RS-enriched cereal foods, whereas the WSD was based on refined grains and a minimal concentration of DF. AX was obtained from whole-grain rye and enzyme-treated wheat bran, and RS was provided as RS type 2[28] from raw potato starch and high-amylose maize starch. The diet allocation sequence was determined by the study coordinators using a simple randomisation method.[29]

The cereal key foods were incorporated into the subjects’ habitual diets (see dietary counselling) and constituted approximately 50% of their total daily energy needs. The diets were isocaloric, as estimated by nutrition labelling. The HCD included experimentally prepared bread rolls (combo rolls) and pancakes (combo pancakes) (Lantmännen R&D, Stockholm, Sweden) that contained RS (7.0 g per roll and 8.4 g per pancake) in the form of high-amylose maize starch (HI-MAIZE260®) (Ingredion Incorporated Inc., Bridgewater, NJ, USA) and AX (6.0 g per roll and 8.4 g per pancake) in form of enzyme-treated wheat bran[30] (DuPont Nutrition and Biosciences ApS, Brabrand, Denmark). RS in the form of raw potato starch (24 g per day) was provided by KMC (Brande, Denmark). Raw potato starch was consumed unheated and dissolved in a smoothie or water. Rye flakes (Lantmännen Cerealia A/S, Vejle, Denmark), rye bread (Lantmännen Schulstad A/S, Hvidovre, Denmark), rye pasta (Il Fornaio, Corte Madera, California, USA) and smoothies (Rynkeby, Ringe, Denmark) were commercially available products.

For the WSD, the subjects received corn flakes and wheat bread (Coop Denmark A/S, Albertslund, Denmark), spelt bread rolls (Lantmännen Unibake, Horsens, Denmark), wheat pasta and pancakes (Lantmännen Cerealia A/S, Vejle, Denmark), and smoothies (Rynkeby, Ringe, Denmark). The key foods were provided at two-week intervals. Due to the different appearances of low and high-fibre key foods, single-blinding of the participants, as described in the original trial protocol, was not feasible. The nutritional compositions of the key foods are shown in Table 1.

**Table 1:** Daily intake of nutritional constituents of the key foods in the Western-style diet (WSD) and the healthy-carbohydrate diet (HCD).

	**WSD**	**HCD**
**Energy (KJ)**^**a**^	5280	4722
**Protein (g) (E%)**	40.4 (13)	31.7 (11)
**Fat (g) (E%)**	17.3 (12)	17.3 (14)
**Digestible carbohydrates (g) (E%)**	225.6 (73)	180.8 (65)
Sugars (glucose, fructose, sucrose) (g)	27.0	25.9
Lactose (g)	2.7	2.4
Digestible starch (g)	195.9	152.5
**Non-digestible carbohydrates (g)**	14.2	59.0
Resistant starch (g)	2.8	20.7
Non-starch polysaccharides (g)	8.5	32.3
Cellulose (g)	1.2	4.2
Arabinoxylan (g)	3.6	16.0
LMW non-digestible carbohydrates (g)	2.9	6.0
Fructan (g)	2.1	5.3
Arabinoxylan oligosaccharides (g)	0.8	0.7
**Lignin (g)**	3.4	5.0
**Dietary fibre (g)**^**b**^**(E%)**	17.6 (3)	64.0 (11)

E%, energy percentage; LMW, low molecular weight.

^a^Calculated as the sum of: 17 kJ per g digestible carbohydrate 8 kJ per g dietary fibre, 17 kJ per g protein and 37 kJ per g fat.

^b^Calculated as the sum of non-digestible carbohydrates and lignin.

### Dietary counselling, energy intake and compliance

A clinical dietician instructed each subject at the beginning of both interventions. Based on individual energy need, a dietary plan was developed to maintain stable body weight and to limit DF except for that obtained from the key foods. The subjects were requested to maintain their regular lifestyles, including physical activity, smoking habits, alcohol intake and medication, throughout the study. The subjects were provided with electronic kitchen scales and checklists of the key food items to ensure dietary adherence. Before and during the diets, the subjects completed food records on three consecutive days, one of which was a weekend day. The macronutrient compositions of the habitual diets were calculated by Master Dietist System version 1.235 (2007) based on the Danish National Food Administration Database. The macronutrient compositions of the intervention diets were calculated as the sum of the compositions of the key foods and what the subjects ingested in addition to the key foods according to the food records. We assumed that the subjects consumed all of the key foods delivered. However, data were missing from seven food records (3 pre-diets, 2 HCD and 2 WSD) due to insufficient registration.

### Recording of gastrointestinal symptoms and stool parameters

An internally validated questionnaire based on verified scoring systems and rating scales[31–34] was used to assess general wellbeing and gastrointestinal symptoms. Five gastrointestinal symptoms (abdominal pain, bloating, rumbling, flatulence and nausea) and their severities as well as general wellbeing and health concerns were scored on visual analogue scales. We also assessed stool parameters, such as bowel movements, consistency, urge and occurrence of mucus. The subjects were instructed to fill in the questionnaires before and after each dietary intervention. In addition, they registered the number and consistency of stools on the same three days as they completed the food records.

### Faecal sampling and handling

Faecal samples were collected using EasySampler® Stool Sample Collection (Alpha Laboratories Ltd, Hampshire, UK) before and at the end of each dietary intervention. The samples were immediately stored at -20°C, and within 24 hours, they were moved to storage at -80°C without being thawed.

### Body weight and fat percentage

Measurements of waist circumference, body weight (TANITA WB-100A CLASS 111), and body fat percentage (Body Fat Monitor F306, OMRON, Hoofddorp, The Netherlands) were assessed before and at the end of each intervention when the subjects were fasting, wearing one layer of light clothes and had just emptied their bladder.

### Chemical analyses

The total RS levels in foods were determined by the AOAC method (AOAC Official Method 2002.02), as described by McCleary & Monaghan[35] and non-starch polysaccharides (NSPs) in foods and faeces, as described by Bach Knudsen[36] except that acid hydrolysis was performed in 2 M H_2_SO_4_ for one hour instead of in 1 M H_2_SO_4_ for two hours. When analysing the two RS sources, raw potato starch and HI-MAIZE260®, with the NSP procedure, we discovered that a fraction of starch in HI-MAIZE260® withstood gelatinization and hydrolysis and was analysed as part of the NSP fraction. To avoid this interference, dimethyl sulphoxide (DMSO) was used to disperse the RS[37] in the NSP procedure. The RS levels estimated with the two methods were designated as RS_ENZ_ and RS_DMSO_, respectively_._ The other analytical methods used to characterise the chemical compositions of the food items have been previously described by Nielsen et al.[15]

The concentrations of faecal SCFAs and other acids including lactic acid were determined by gas-liquid chromatography (HP-6890 Series Hewlett Packard Palo Alto, CA) according to Jensen et al.[38] The total SCFA concentration was calculated as the sum of the formic acid, acetate, propionate, isobutyrate, butyrate, isovaleric and valeric acid concentrations, and the branched-chain fatty acid (BCFA) concentration was calculated as the sum of the isobutyrate and isovaleric acid concentrations.

### Bacterial genomic DNA extraction and 16S rRNA gene sequencing

Genomic DNA was extracted in duplicate from four faecal samples per subject (before and after WSD and HCD consumption) using a QIAamp Fast DNA Stool Mini Kit (Qiagen, Hilden, Germany). Approximately 200 mg was chipped from each sample without thawing and placed directly into sterile 2 ml tubes containing 16 ml QIAamp InhibitEx buffer and 300 mg of 0.1 mm diameter zirconia/silica beads (Biospec Products, Bartlesville, Oklahoma, USA). The samples were shaken twice for 1 min each time at 6.5 m/s in a MP FastPrep-24 tissue and cell homogeniser (MP Bio, Santa Ana, California, USA) prior to completing the DNA extractions and purifications, according to the manufacturer’s instructions for pathogen detection. DNA concentration was measured using a NanoDrop spectrophotometer (Thermo Scientific, Wilmington, Delaware, USA) and diluted to 20 ng/μL. Equal volumes of duplicate DNA extractions from each faecal sample were combined prior to PCR amplification.

The 16S rRNA V4 region was amplified by PCR using the barcoded F515 forward primer and R806.[39, 40] Amplification was performed using Ex Taq DNA Polymerase (TaKaRa, Otsu, Japan) for 30 cycles of 94°C for 45 seconds, 54°C for 60 seconds, and 72°C for 30 seconds. PCR products were purified using a Wizard® SV Gel and PCR Clean-Up System (Promega, Madison, Wisconsin, USA), and the pooled amplicons were sequenced with an Illumina MiSeq at the Genome Center DNA Technologies Core, University of California, Davis, California, USA.

Raw Illumina FASTQ sequences were analysed using Quantitative Insights Into Microbial Ecology (QIIME) software package version 1.8.0.[41] Demultiplexing and quality filtering were performed with the default settings, except that a minimum average quality score of 30 was used for quality filtering instead of the default score of 25, and the reverse primer sequences were removed. The open-reference OTU picking strategy in QIIME was used to select operational taxonomic units (OTUs) with 97% sequence identity to sequences in Greengenes (release 13_8) database[42] and to each other, according to the UCLUST algorithm.[43] OTUs similar to chloroplasts or those found in very low abundance (< 0.005%)[44] were removed from the OTU table prior to further analysis. The Chao1 diversity index, Phylogenetic Diversity Whole Tree,[45] and observed species were determined for increasing numbers of randomly sampled sequences per sample to generate alpha diversity rarefaction curves. The curves became asymptotic at 12,000 sequences per sample and therefore, this number of randomly sampled DNA sequences was used for calculation of the weighted UniFrac metric[46] in QIIME.

### Statistical methods

Prior to conducting the study, a sample size was calculated based on an expected increase in the faecal butyrate concentration after HCD consumption compared with that after WSD consumption.

Based on a Danish study population treated with approximately 18 g of dietary fibre (*Plantago ovata* seeds) for 4 weeks,[47] we anticipated that the standard deviation of the mean difference in butyrate concentrations between the diets would be 7.5 mmol/l and that the minimal relevant difference between means would be 7.5 mmol/l–thus we used a standardized minimal relevant difference of 1 in a crossover design. The power calculation was done using software developed by Schoenfeld[48] and by formulas given by Julious et al.[49] The total number of subjects needed to obtain a statistical power of 90% for a type I error of 5% in a crossover trial was calculated to be 13. The anticipated dropout rate was set to 33%.

Descriptive statistics are given as the median with the interquartile range (IQ). Analysis of variance for repeated measurements was performed to examine faecal SCFA and carbohydrate residue responses using subject, diet, period, and baseline values as covariates. These data were log-transformed to obtain a Gaussian distribution. Model validation was performed by inspection of Bland-Altman plots to check if the mean and standard deviation of the differences were constant throughout the range[50] and by probability plots of the residuals. Additional statistical analyses of the total SCFA, acetate, propionate and butyrate concentrations were carried out, excluding the two participants receiving the anthelmintic treatment, which had no effect on the results.

The dietary data, body measurements, data from the questionnaires and differences from the baselines to the end of the diets were compared with the two-tailed paired t-test. Model validation was performed by Bland-Altman plots and probability plots of the differences. Wilcoxon signed-rank tests were performed for comparison of the DF intakes, protein intakes, faecal characteristics and gastrointestinal symptoms, because the assumptions for the two-tailed t-test were not feasible. These statistical analyses were conducted using STATA/IC 12.1 (StataCorp, College Station, Texas, USA), and Graph Pad Prism 6 (Graph Pad Software, Inc., La Jolla, California, USA) was used to generate graphical elements.

Differences in UniFrac distances between samples from different experimental groups were tested by the Wilcoxon signed rank test followed by Bonferroni correction of the resulting p-values. Bacterial taxa that were present at greater than 0.1% abundance in at least 20% of the samples from 18 subjects who collected faecal samples throughout the entire study period were evaluated using R[51] with the paired Wilcoxon signed-rank test followed by Benjamini and Hochberg false discovery rate adjustment of the p-values. Alpha diversity measurements were compared in the same manner as the bacterial taxa comparisons. Differences were regarded as significant at a *P* < 0.05.

## Results

### Study population

**Fig 1:**
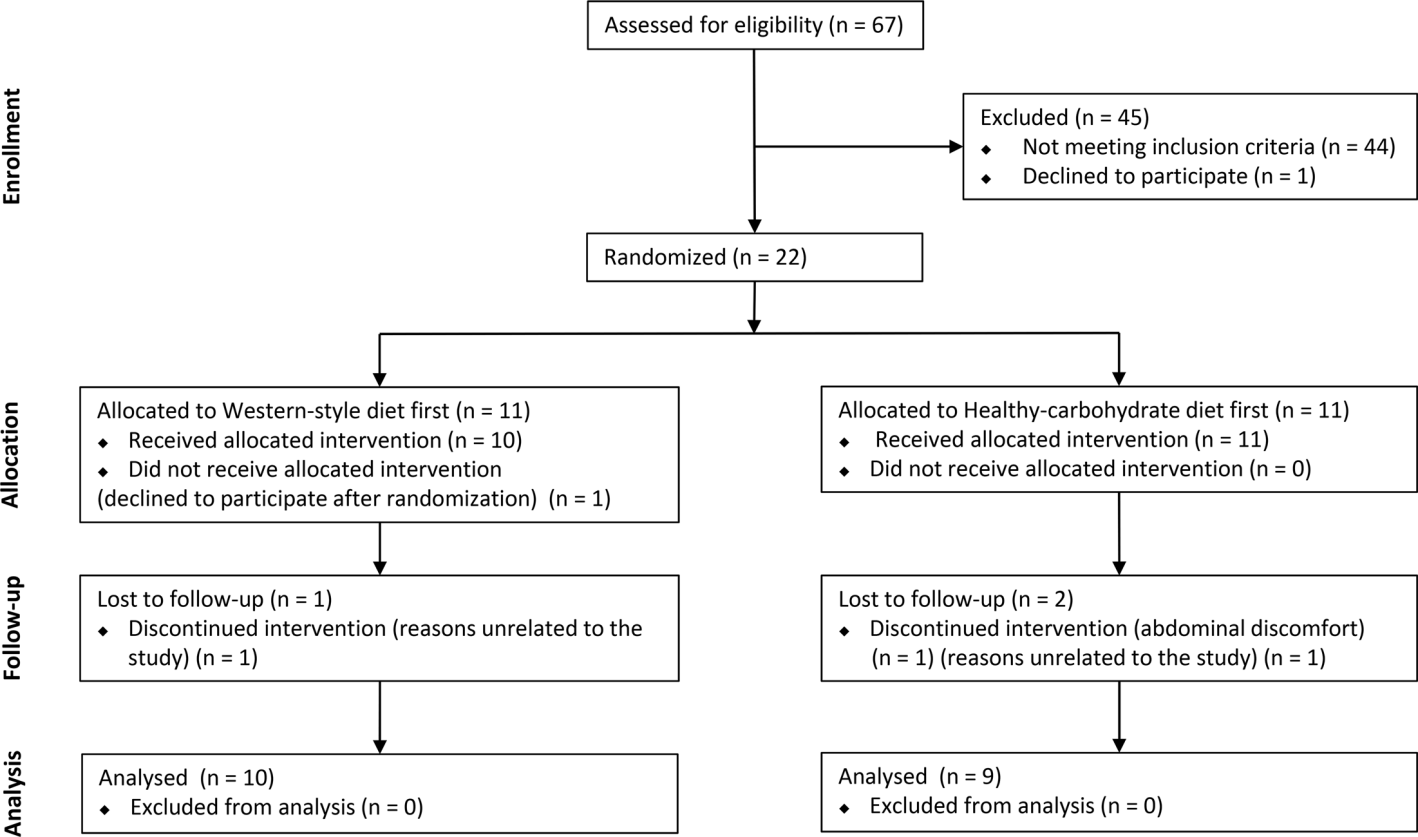
Subject flow.

A total of 67 adults were screened and 22 met the inclusion criteria and were randomized between June 2012 and January 2013 (Fig 1) (S2 Information). Nineteen subjects completed the study. Two subjects withdrew for reasons unrelated to the study, and one left the study during the first week of the HCD because of abdominal discomfort. Seven subjects were treated with antihypertensive drugs, and seven were treated with cholesterol-lowering medication at consistent doses throughout the study. Two subjects received treatment with mebendazole for 4 weeks (HCD) due to pinworm infection. The baseline characteristics and habitual macronutrient intakes of the subjects are shown in Table 2.

**Table 2:** Baseline characteristics and habitual macronutrient intakes.

	**Median**	**Interquartile range**	**Total range**
**Age (years)**	60	48–67	39–75
**BMI (kg/m**^**2**^**)**	30.6	29.3–35.2	25.9–41.0
**Waist (cm)**	106	103–116	89–130
**Body fat (%)**	33	30–39	25–49
**Energy intake (KJ/day)**^**a**^	7812	6920–8845	4600–13991
**Protein (g)**^**a**^	79	69–91	53–140
**Fat (g)**^**a**^	70	56–94	32–160
**Total carbohydrates (g)**^**a**^	214	172–234	130–370
**Digestible carbohydrates (g)**^**a**^	197	154–210	117–335
**Dietary fibre (g)**^**a**^	18	14–25	8–35

^a^Based on dietary records from first run-in (n = 19).

### Diets, energy intake and anthropometric data

There were no significant differences in the energy intake (*P* = 0.75) of the subjects or their consumption of protein (*P* = 0.91), fat (*P* = 0.82), carbohydrates (*P* = 0.36) or DF (*P* = 0.12) during the two run-in periods. The baseline characteristics and habitual macronutrient intake from first run-in period is shown in Table 2. The DF concentration in the key foods was 64 g/day for the HCD and 18 g/day for the WSD (Table 1). Thus, the subjects increased their total DF intake from a median of 18 g/day (IQ 14–25 g/day) at baseline (Table 2) to a median of 68 g/day (IQ 66–75 g/day) during the HCD (*P* < 0^.^001) (Table 3), whereas it remained stable, with a median of 21 (IQ 18–22) g/day during the WSD (*P* = 0.38) (Table 3). The key foods provided a 7-fold difference in the RS concentration between the WSD (2.8 g/day) and HCD (20.7 g/day) and an almost 4.5-fold difference in the AX concentration between the WSD (3.6 g/day) and HCD (16.0 g/day) (Table 3).

**Table 3:** Composition of total dietary intake during the Western-style diet (WSD) and the healthy-carbohydrate diet (HCD).

	WSD (n = 17)^a^			HCD (n = 17)^a^			WSD vs. HCD^b^
	Median	IQ range	Total range	Median	IQ range	Total range	*P-value*
**Energy (KJ)**	9217	8450–10611	6855–14516	8412	7899–11228	6566–16799	0.97
**Total CHOs (g)**	300	271–346	260–479	325	292–375	269–603	0.17
**Digestible CHOs (g)** ^**c**^	281	253–322	242–434	244	224–334	204–487	0.07
**Dietary fibre (g)**	21	18–22	17–45	68	66–75	65–116	0.0007
**Protein (g)**	96	85–111	53–153	83	74–105	65–222	0.21
**Fat (g)**	62	54–82	36–105	63	42–76	37–148	0.50

IQ, interquartile; CHOs, carbohydrates.

^a^Calculated as the sum of macronutrition according to food records and the daily ration of key foods, as determined by chemical analysis.

^b^Differences between the interventions compared by paired t-test, except for dietary fibre and protein, which were compared by Wilcoxon signed-rank test.

^c^Calculated as: (total carbohydrates–dietary fibre according to food records) + (digestible carbohydrates determined by chemical analysis).

The energy content was 12% higher in the WSD key foods than in the HCD key foods (Table 1) because of the unexpectedly high protein content of the WSD bread and pasta compared with the HCD equivalents (S1 Table). The differences in the energy and nutritional constituents of the key foods were diminished when the total dietary intake was taken into account (Table 3). The median energy need of the subjects was determined to be 10,605 kJ (total range 7,992–13,402 kJ) per day, and the energy from the key foods in the HCD and WSD provided the subjects with 44% and 50% of their calculated median energy needs, respectively. The subjects’ body weights, waist circumferences and body fat percentages remained unchanged throughout the study (S2 Table).

### Faecal characteristics and gastrointestinal symptoms

Bowel movements over the three-day registration period rose from a median of 4 (total range 1–8) during the WSD to 5 (total range 2–13) (*P* < 0^.^01) during the HCD. The faecal consistency differed significantly (*P* = 0.02) between the two interventions. Six subjects reported constipation during the WSD compared with one subject during the HCD. Constipation difficulties were addressed by guidance on increased water intake and exercise, and no laxatives were necessary.

Flatulence (*P* < 0.001) and stomach rumbling (*P* = 0.05) were increased during the HCD, but no other abdominal symptoms or adverse effects were reported. The diets were well tolerated and did not influence the subjects’ health concerns or abdominal symptom severity. Self-reported wellbeing was rated as moderately higher during the HCD compared with the WSD (*P* = 0.05).

### Faecal carbohydrate residues

There were no differences in the faecal concentrations of carbohydrate residues (dry matter basis (DM)) during the pre-WSD and pre-HCD periods (Table 4). Consumption of the WSD was followed by a decline in total faecal DF from 17.2% DM to 12.3% DM (*P* = 0.01), whereas the opposite was the case for the HCD, which resulted in an increase from 18.4 to 24.2% DM (*P* = 0.002) (Table 4). Thus, faecal DF was 2 fold (95% CI 2-3-fold) (*P* < 0.0001) higher after HCD consumption compared with WSD consumption. In addition, the HCD increased RS residues from 2.3% DM to 3.0% DM compared with 0.8% DM after the WSD (*P* = 0.003), while AX residues were increased to 8.0% DM following the HCD compared with 3.3% DM after the WSD (*P* < 0.0001).

**Table 4:** Faecal carbohydrate residues (% of dry matter) during the pre-periods and after consumption of the healthy-carbohydrate diet (HCD) compared with the Western-style diet (WSD).

	Pre-WSD (n = 19)		WSD (n = 19)		Pre-HCD (n = 19)		HCD (n = 19)			**HCD vs. WSD**
	Mean	95% CI	Mean	95% CI	Mean	95% CI	Mean	95% CI	SEM	*P-value*
RS_DMSO_^a^	2.1	1.2–2.9	0.8	0.3–1.2	2.3	0.7–3.8	3.0	1.4–4.7	-	0.003
NSPs	15.1		11.6		16.1		21.2		1.29	< 0.0001
Cellulose	4.2		3.2		4.1		4.7		0.37	0.002
Arabinoxylan	4.6		3.3		4.7		8.0		0.55	0.0001
Arabinose	2.1		1.5		2.2		3.8		0.26	< 0.0001
Xylose	2.5		1.8		2.5		4.1		0.30	< 0.0001
A:X ratio	0.84		0.86		0.88		0.93		0.04	0.29
Rhamnose^a^	0.5	0.5–0.6	0.6	0.5–0.7	0.5	0.5–0.6	0.6	0.5–0.7	-	0.07
Fucose^a^	0.2	0.2–0.2	0.2	0.2–0.3	0.2	0.2–0.2	0.2	0.2–0.2	-	0.25
Mannose	0.6		0.6		0.6		0.5		0.05	0.32
Galactose	1.3		1.3		1.3		1.8		0.08	< 0.0001
Glucose^a^	3.1	2.2–3.9	1.9	1.4–2.3	3.9	2.0–5.8	4.5	3.1–5.8	-	0.0001
Uronic acid	0.8		0.7		0.8		1.0		0.07	0.002
Dietary fibre^a^	17.2	14.5–19.8	12.3	10.2–14.5	18.4	14.3–22.6	24.2	20.6–27.9	-	< 0.0001

A:X ratio, arabinose to xylose ratio; CI, confidence intervals; NSPs, non-starch polysaccharides; RS_DMSO_, resistant starch; SEM, standard error of the mean (ANOVA for repeated measurements using subject, diet, period and baseline values as covariates).

^a^Data were logarithmically transformed before data analysis and therefore, 95% CI are given.

### Faecal microbial composition

16S rRNA gene amplicon sequencing was performed to identify the microbial compositions in faecal samples collected at baseline and at the end of the interventions. An average of 59,113 sequencing reads was obtained for each sample after quality filtering, including an average of 863 OTUs per sample. The number of observed species (alpha-diversity) was significantly lower in the stools collected after the HCD (an average of 615 species) compared with those collected after the WSD (675 species) (*P* < 0.0001). This difference in species richness was in agreement with the Chao1 (*P* < 0.05) and phylogenetic diversity (*P* < 0.005) alpha-diversity metrics.

**Fig 2:**
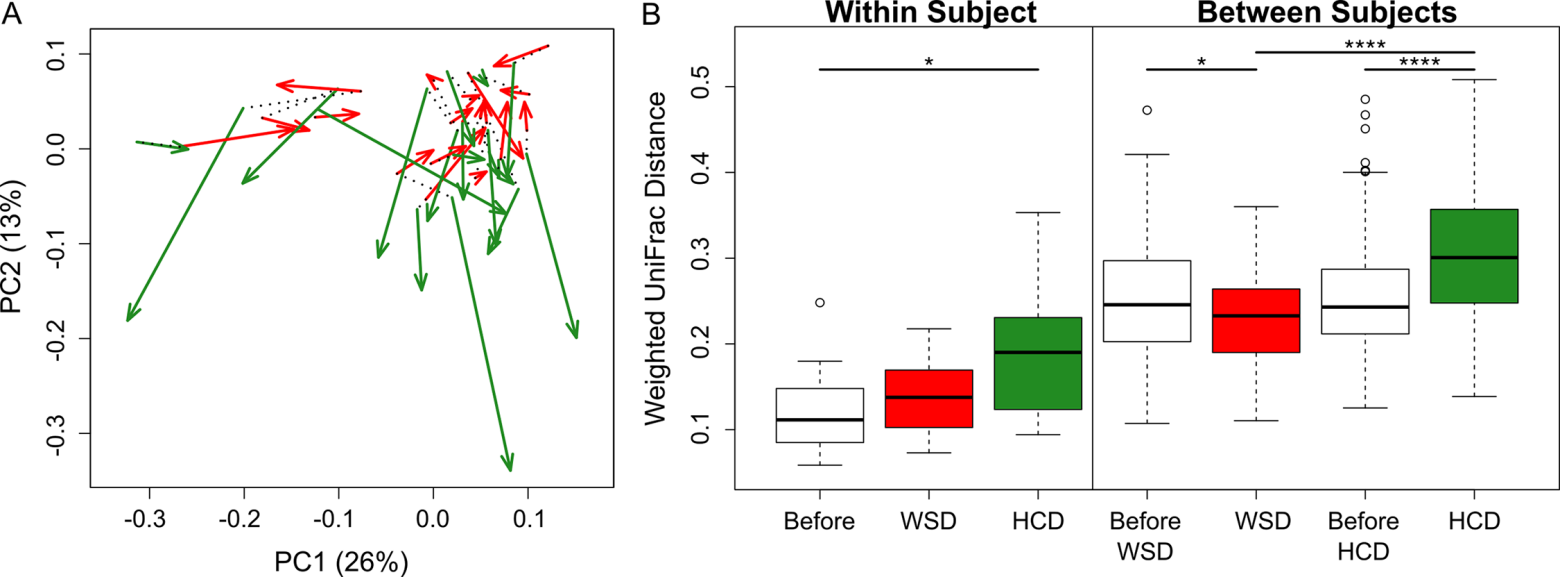
A Principal coordinate analysis of weighted UniFrac values between faecal microbial communities. The green and red arrows represent bacterial communities before and at the end of healthy-carbohydrate diet (HCD) and Western-style diet (WSD) consumption, respectively. The dotted lines connect the microbiota present during consumption of the two baseline diets for each individual. **B Box plot of the weighted UniFrac distances.** Intra-individual microbiota distances are shown on the left, and inter-individual distances are shown on the right. * *P*< 0.05, **** *P*< 0.0001 (Wilcoxon signed-rank test).

The HCD also resulted in a significant change in the beta-diversity of the faecal microbiota, as shown by principal coordinates analysis of the weighted UniFrac distance metric (Fig 2A). The effects of the HCD were observed both within and between subjects (Fig 2B). Specifically, the microbiota were more heterogeneous, as indicated by the larger weighted UniFrac distances after HCD consumption compared with the baseline values for individual subjects (Fig 2B, “Within Subjects”), as well as for all subjects combined (Fig 2B, “Between Subjects”).

**Fig 3:**
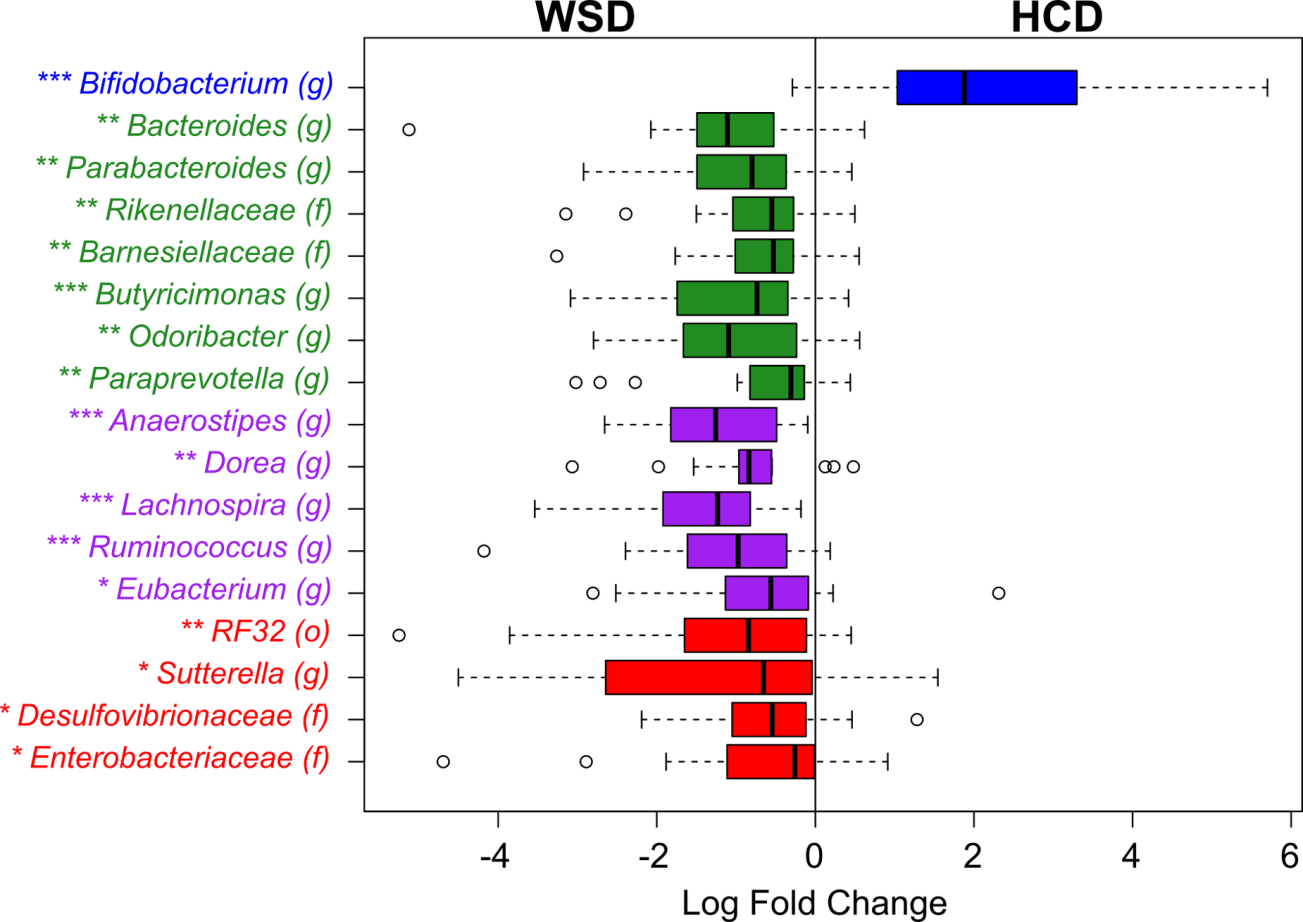
Box plot of the log_2_-transformed fold-changes in the relative abundances of taxa in the faeces collected at the end of consumption of the healthy-carbohydrate diet (HCD) compared with that of the Western-style diet (WSD). Only taxa that were significantly affected by diet (*P* < 0.05) are shown. Family, order, and genus distinctions are represented by (f), (o), and (g), respectively. The boxes and text are coloured according to the phyla as follows: *Actinobacteria* (blue), *Bacteroidetes* (green), *Firmicutes* (purple), and *Proteobacteria* (red). * *P* < 0.05, ** *P* < 0.01, *** *P* < 0.001 (Wilcoxon signed-rank test).

The proportion of *Bifidobacterium* in the faecal microbiota was significantly increased after HCD consumption compared with that detected after WSD consumption (Fig 3). Non-significant increases were also observed in *Lactobacillus*, *Clostridium*, and *Akkermansia*. Conversely, the HCD resulted in reductions in the proportions of the *Bacteroidetes* genera *Bacteroides*, *Parabacteroides*, *Butyricimonas*, *Odoribacter*, and *Paraprevotella* (Fig 3). The proportions of five genera in the *Firmicutes* phylum were also lower following consumption of the HCD, including certain *Ruminococcus* species (Fig 3). These *Ruminococcus* species are members of the *Lachnospiraceae* family and are not related to *Ruminococcus bromii*, another bacterial species associated with consumption of DF.[14, 17–19] The proportions of certain members of *Proteobacteria* were also decreased following the HCD compared with the WSD, including *Sutterella* and members of the *Desulfovibrionaceae* family.

### Faecal SCFAs and lactic acid

**Fig 4:**
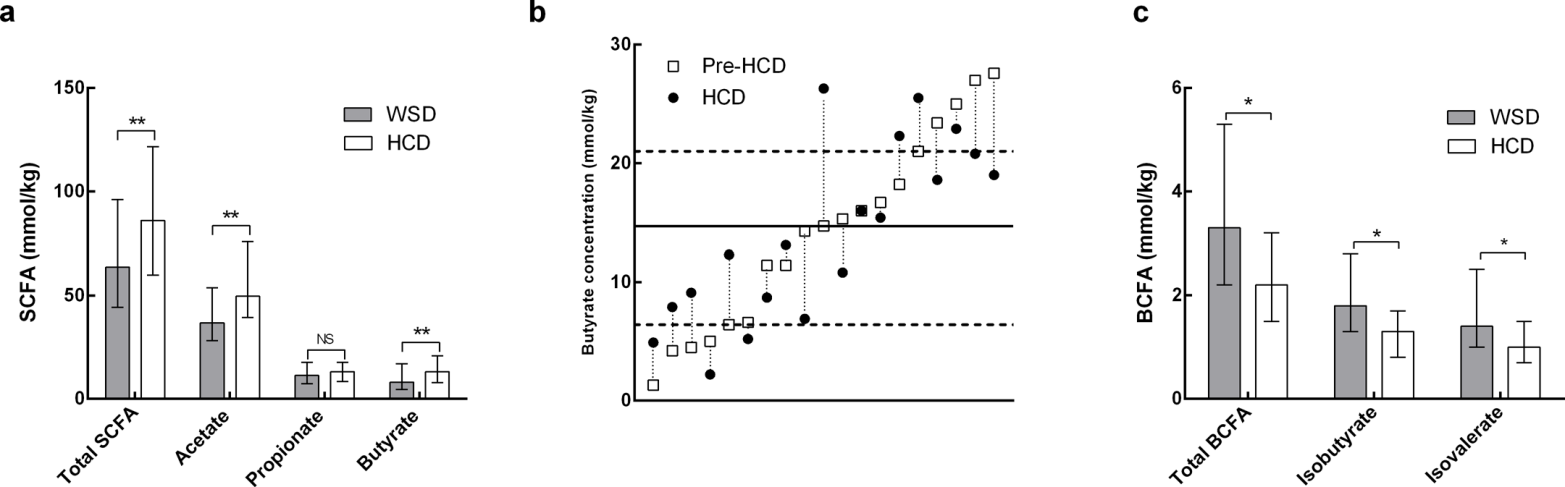
Faecal short-chain fatty acid (SCFA) and branched-chain fatty acid (BCFA) concentrations. **A, C** Differences between Western-style diet (WSD) and healthy-carbohydrate diet (HCD) concentrations are indicated by NS; non significant, *P < 0.05 and **P< 0.01 (ANOVA for repeated measurements using subject, diet, period and baseline values as covariates). The values are the medians with interquartile ranges (n = 19) as the data were log-transformed. **B** Individual faecal butyrate concentration before (Pre-HCD) and after HCD. The solid line represents the median pre-HCD concentration and the dashed lines indicate the interquartile range of the pre-HCD concentrations.

The SCFA concentrations during the two run-in periods were similar. The diets caused significant differences in SCFA concentrations, as illustrated in Fig 4A. During the WSD (from weeks 0 to 4) both acetate and butyrate declined 19% (95% CI 5–30%) (*P* = 0.01) and 37% (95% CI 22–50%) (*P* < 0.001), respectively. Conversely, acetate (*P* = 0.12) and butyrate (*P* = 0.68) remained unchanged during the HCD. A large range was observed in the pre-HCD concentrations, from 1.3 to 27.6 mmol/kg. The butyrate concentration increased in response to the HCD in half of the subjects, mainly those with the lowest pre-HCD levels, whereas the subjects with high pre-HCD concentrations experienced a decline in the concentration after HCD consumption (Fig 4B). The concentration of lactic acid was not affected by the diet (*P* = 0.18).

The total concentration of branched-chain fatty acids declined 30% (95% CI 3–50%) (*P* = 0.03), that of isobutyrate decreased 28% (95% CI 1–48%) (*P* = 0.05) and that of isovalerate decreased 21% (95% CI 6–52%) (*P* = 0.03) after the HCD compared with the WSD (Fig 4C). The caproic acid concentration rose slightly (from a median of 0.5 to 1.1 mmol/kg (*P* = 0.001)) during the HCD. The concentrations of formic acid, valeric acid, heptanoic acid and succinic acid concentrations remained unchanged, and those of sorbic acid, benzoic acid and hippuric acid were all below the detection levels.

## Discussion

In this 4-week, randomised, crossover, dietary intervention study of individuals with MetS, we found that a diet rich in RS and AX modified the gut microbiome and increased the concentration of faecal SCFAs in general and those of butyrate and acetate in particular compared with a low-fibre diet.

The HCD conferred a significant decrease in bacterial species richness in all individuals. This finding corroborates that of a previous study reporting that a diet high in RS is associated with decreased bacterial diversity relative to other diets.[52] However, we also found that HCD consumption increased the inter- and intra-individual variation of the intestinal microbiota. This finding indicates that the enrichment of specific populations of bacteria in the intestine by DF is both diet- and subject-dependent.[18, 19] This effect of DF was significant because unlike other human dietary intervention studies,[52, 53] the intra-individual differences in the intestinal microbiota were typically as large as those found between subjects. This outcome is probably due to the strong selection of certain bacteria by DF of the taxa that were already present in the intestines of each individual prior to the HCD intervention.[19] In addition to dietary factors, the intestinal microbiota is influenced by e.g. decreased diversity in elderly subjects, immunological factors, antibiotic treatment, genetic heritage and microbial exposition at birth.[9] The range from 39 to 75 years of age in the subjects may contribute to the between- subject differences.

The predominant change in the faecal microbiota with the introduction of the HCD was the enrichment of members of *Bifidobacterium*, a genus regarded to be beneficial to human health.[54] The capacity of DF to promote *Bifidobacterium* has been found in many studies.[14, 15, 55] *Bifidobacterium* is a saccharolytic bacterium that produces acetate and lactic acid as a result of fermentative growth.[56, 57] Hence, the increased faecal acetate concentration observed with HCD consumption may have been the result of stimulation of *Bifidobacterium* growth and metabolism by DF in the gut. Acetate production by *Bifidobacterium* confers protection against gastrointestinal pathogens[58, 59] and it may be consumed by butyrate-producing bacteria in the gut.[60, 61] An *in vitro* study has also found that up to 90% of produced butyrate is derived from acetate.[62] The explanation why we did not find a diet effect on lactic acid may be that it is readily fermented whereby it also acts as a precursor for butyrate synthesis.[63] Therefore, the increased faecal butyrate concentration is most likely the result of increased acetate and lactic acid produced by *Bifidobacterium* rather than stimulation of butyrate-producing microorganisms.

Conversely, *Firmicutes*, *Bacteroidetes* and *Proteobacteria* were significantly reduced by the HCD compared with the WSD. WSD consumption, on the other hand, enhanced the proportions of *Bacteroides*, *Sutterella* and *Ruminococcus*. The specific *Ruminococcus* species enriched by the WSD are members of the *Lachnospiraceae* family, which includes species previously associated with inflammatory bowel disease.[64]

In the present study, we were unable to differentiate between the contribution of AX and that of RS to the enhanced SCFA production. AX derived from whole-grain rye has been demonstrated to cause a significant increase in the butyrate concentration in humans,[3] and AX derived from whole-grain rye and enzyme-treated wheat bran has been found to be superior to RS type 2 with regard to butyrate and acetate production in pigs.[15, 30] However, a concentrate of AX from wheat has been demonstrated not to affect the colonic butyrate concentration or the caecal digesta butyrate pool in pigs.[65] The reasons for these differences in butyrate response among these studies cannot be determined with certainty but may potentially be related to the manner by which AX was provided, for example, as a concentrate from wheat[66] or as part of a whole-grain matrix.[3, 16] Fermentation of an AX concentrate can be expected to be rapid and accompanied by a pH drop, mainly in the caecum,[66] whereas the fermentation of AX from a whole-grain matrix will be slower, with pH drops not only in the caecum but also in the proximal and mid colon.[20] In the present study, this situation was even more complex because the RS provided from raw potato starch was degraded more rapidly than that from high-amylose corn starch, which was degraded at a slower rate at more distal locations.[20] Based on the results of Nielsen et al.[15], we speculate that the RS from high-amylose corn starch was responsible for the higher RS levels found in the faeces after HCD consumption.

Surprisingly, the diet-induced differences in the SCFA concentrations observed in this study were mainly due to declining SCFA concentrations during the WSD, despite the significantly increased consumption of total DF in the HCD compared with that in the WSD and at baseline. Furthermore, the faecal DF, RS and AX residues increased during the HCD and declined during the WSD, indicating good dietary adherence during the study. One possible explanation for the changes in the SCFA concentrations observed during the interventions might be that the subjects had a higher habitual intake of DF than that registered in the food records because rye bread in particular is a normal part of the Danish diet. In addition, food data obtained from food records may be underreported.[66] Still, Jonsdottir et al.[67] found a mean DF intake of 22 g/d in a Nordic population with MetS, which is similar to the reported baseline intake among the subjects reported here. A second explanation may be that the subjects were instructed to limit their intake of fibre-rich fruits, nuts and vegetables during the interventions, and it is likely that such limited intake contributed to their changes in intestinal microbial environment and SCFA production. A third explanation may be that we underestimated the effect of the HCD on total colonic SCFA production because we did not consider potentially increased faecal output from DF. Recently, it has been shown that diets rich in RS or in AX increase both the production and faecal output of total SCFAs, particularly acetate and butyrate, in pigs.[15] We did find a significant increase in bowel movements during the HCD, pointing to increased faecal output.

A noteworthy finding was the decreased concentration of BCFAs in the faeces during the HCD. This finding indicates reduced protein fermentation, which counteracts the accumulation of potentially harmful metabolites.[68] *Bacteroides*, an abundant human gut resident[69] capable of proteolytic fermentation[56] and BCFA production,[70] was down-regulated by the HCD, which corroborates these results. Cummings et al.[71] have also reported decreased BCFAs after RS-enriched diet consumption. Consumption of a diet rich in AX has also been shown to lower caecal digesta *p*-cresol, indicating reduced protein fermentation.[65]

A limitation of the present study is that the dietary compositions were based on nutrition labelling, but chemical food analyses performed post hoc disclosed that the key foods had a higher protein content in the WSD than in the HCD, which could have resulted in differences in the BCFAs. Nevertheless, these differences were evened out when total dietary intake was taken into account, causing the difference in total protein intake between the groups to lose significance, and only DF intake was significantly different in favour of the HCD.

In conclusion, the results of our study support the hypothesis that high intake of both RS and AX is capable of changing the intestinal microbiota and SCFA production in subjects with MetS in contrast with a low-fibre WSD. Most distinctly, *Bifidobacterium* was clearly enriched by the HCD, which was in strong agreement with the increased faecal acetate concentration. Also, dysbiotic changes observed during the WSD emphasise the need for balanced diets, including DF from various sources. However, long-term randomised controlled intervention studies are needed to investigate the effects of DF on the microbiota and SCFA production in a more continuous setting. In addition, further attempts to compare the impacts of different DFs on metabolic risk factors and intestinal mucosal function are needed.

## Supporting Information

S1 Information Study protocol. English translation and the original version in Danish.
(DOCX)

S2 Information Consort checklist. (DOC)

S1 Table Nutritional composition of the key foods on a dry matter (DM) basis for the healthy-carbohydrate diet (HCD) and Western-style diet (WSD). (DOCX)

S2 Table Bodyweight, body fat percentage and waist circumference at run-in and at the end of the healthy-carbohydrate diet (HCD) and Western-style diet (WSD). Values are presented as medians and interquartile ranges in brackets.
(DOCX)

## Abbreviations

AX: arabinoxylan
BCFAs: branched-chain fatty acids
DF: dietary fibre
DM: dry matter
HCD: healthy-carbohydrate diet
MetS: metabolic syndrome
NSPs: non-starch polysaccharides
RS: resistant starch
RS_DMSO_: resistant starch estimated with a method using dimethyl sulphoxide
RS_enz_: resistant starch estimated with an enzyme-containing kit
SCFAs: short-chain fatty acids
WSD: Western-style diet

## References

[1] Hartvigsen ML, Gregersen S, Laerke HN, Holst JJ, Bach Knudsen KE, Hermansen K. Effects of concentrated arabinoxylan and beta-glucan compared with refined wheat and whole grain rye on glucose and appetite in subjects with the metabolic syndrome: a randomized study. European journal of clinical nutrition. 2014;68(1):84–90. 10.1038/ejcn.2013.236 24253758

[2] Kovatcheva-Datchary P, Arora T. Nutrition, the gut microbiome and the metabolic syndrome. Best practice & researchClinical gastroenterology. 2013;27(1):59–72. 10.1016/j.bpg.2013.03.017 23768553

[3] McIntosh GH, Noakes M, Royle PJ, Foster PR. Whole-grain rye and wheat foods and markers of bowel health in overweight middle-aged men. The American Journal of Clinical Nutrition. 2003;77(4):967–74. 12663299 10.1093/ajcn/77.4.967

[4] Le Chatelier E, Nielsen T, Qin J, Prifti E, Hildebrand F, Falony G, et al Richness of human gut microbiome correlates with metabolic markers. Nature. 2013;500(7464):541–6. 10.1038/nature12506 23985870

[5] Haenen D, Zhang J, da Silva CS, Bosch G, van der Meer IM, van Arkel J, et al A Diet High in Resistant Starch Modulates Microbiota Composition, SCFA Concentrations, and Gene Expression in Pig Intestine. The Journal of nutrition. 2013 10.3945/jn.112.169672 23325922

[6] Yamashita H, Fujisawa K, Ito E, Idei S, Kawaguchi N, Kimoto M, et al Improvement of obesity and glucose tolerance by acetate in Type 2 diabetic Otsuka Long-Evans Tokushima Fatty (OLETF) rats. Bioscience, biotechnology, and biochemistry. 2007;71(5):1236–43. JST.JSTAGE/bbb/60668 [pii]. 17485860 10.1271/bbb.60668

[7] Lin HV, Frassetto A, Kowalik EJ Jr., Nawrocki AR, Lu MM, Kosinski JR, et al Butyrate and propionate protect against diet-induced obesity and regulate gut hormones via free fatty acid receptor 3-independent mechanisms. PloS one. 2012;7(4):e35240 10.1371/journal.pone.0035240 22506074 PMC3323649

[8] Gao Z, Yin J, Zhang J, Ward RE, Martin RJ, Lefevre M, et al Butyrate improves insulin sensitivity and increases energy expenditure in mice. Diabetes. 2009;58(7):1509–17. 10.2337/db08-1637 19366864 PMC2699871

[9] Scott KP, Gratz SW, Sheridan PO, Flint HJ, Duncan SH. The influence of diet on the gut microbiota. Pharmacological research. 2013;69(1):52–60. Epub 2012/11/14. 10.1016/j.phrs.2012.10.020 . 23147033

[10] Lattimer JM, Haub MD. Effects of dietary fiber and its components on metabolic health. Nutrients. 2010;2(12):1266–89. 10.3390/nu2121266; 10.3390/nu2121266 22254008 PMC3257631

[11] Starch Resistant. Proceedings for the 2nd plenary meeting of EURESTA: European FLAIR Concerted Action No. 11 on physiological implications of the consumption of resistant starch in man. Crete, 29 May-2 June 1991. European journal of clinical nutrition. 1992;46 Suppl 2:S1–148. 1425538

[12] Weaver GA, Krause JA, Miller TL, Wolin MJ. Cornstarch fermentation by the colonic microbial community yields more butyrate than does cabbage fiber fermentation; cornstarch fermentation rates correlate negatively with methanogenesis. The American Journal of Clinical Nutrition. 1992;55(1):70–7. 1309475 10.1093/ajcn/55.1.70

[13] van Munster IP, Tangerman A, Nagengast FM. Effect of resistant starch on colonic fermentation, bile acid metabolism, and mucosal proliferation. Digestive diseases and sciences. 1994;39(4):834–42. 8149850 10.1007/BF02087431

[14] Martinez I, Kim J, Duffy PR, Schlegel VL, Walter J. Resistant starches types 2 and 4 have differential effects on the composition of the fecal microbiota in human subjects. PloS one. 2010;5(11):e15046 10.1371/journal.pone.0015046 21151493 PMC2993935

[15] Nielsen TS, Laerke HN, Theil PK, Sorensen JF, Saarinen M, Forssten S, et al Diets high in resistant starch and arabinoxylan modulate digestion processes and SCFA pool size in the large intestine and faecal microbial composition in pigs. The British journal of nutrition. 2014:1–13. S000711451400302X [pii]. 10.1017/S000711451400302X 25327182

[16] Tachon S, Zhou J, Keenan M, Martin R, Marco ML. The intestinal microbiota in aged mice is modulated by dietary resistant starch and correlated with improvements in host responses. FEMS microbiology ecology. 2013;83(2):299–309. 10.1111/j.1574-6941.2012.01475.x 22909308

[17] Abell GC, Cooke CM, Bennett CN, Conlon MA, McOrist AL. Phylotypes related to Ruminococcus bromii are abundant in the large bowel of humans and increase in response to a diet high in resistant starch. FEMS microbiology ecology. 2008;66(3):505–15. 10.1111/j.1574-6941.2008.00527.x 18616586

[18] Ze X, Duncan SH, Louis P, Flint HJ. Ruminococcus bromii is a keystone species for the degradation of resistant starch in the human colon. The ISME journal. 2012;6(8):1535–43. 10.1038/ismej.2012.4 22343308 PMC3400402

[19] Walker AW, Ince J, Duncan SH, Webster LM, Holtrop G, Ze X, et al Dominant and diet-responsive groups of bacteria within the human colonic microbiota. Isme j. 2011;5(2):220–30. Epub 2010/08/06. 10.1038/ismej.2010.118 ; PubMed Central PMCID: PMCPmc3105703. 20686513 PMC3105703

[20] Saulnier L, Sada P-E, Branlard G, Charmet G, Guillon F. Wheat arabinoxylans: Exploiting variation in amount and composition to develop enhanced varieties. Journal of Cereal Science. 2007;46(3):261–81.

[21] Bach Knudsen KE, Serena A, Kjaer AK, Jorgensen H, Engberg R. Rye bread enhances the production and plasma concentration of butyrate but not the plasma concentrations of glucose and insulin in pigs. The Journal of nutrition. 2005;135(7):1696–704. 135/7/1696 [pii]. 15987852 10.1093/jn/135.7.1696

[22] Neyrinck AM, Possemiers S, Druart C, Van de Wiele T, De Backer F, Cani PD, et al Prebiotic effects of wheat arabinoxylan related to the increase in bifidobacteria, Roseburia and Bacteroides/Prevotella in diet-induced obese mice. PloS one. 2011;6(6):e20944 10.1371/journal.pone.0020944 21695273 PMC3111466

[23] Lappi J, Salojarvi J, Kolehmainen M, Mykkanen H, Poutanen K, de Vos WM, et al Intake of whole-grain and fiber-rich rye bread versus refined wheat bread does not differentiate intestinal microbiota composition in Finnish adults with metabolic syndrome. The Journal of nutrition. 2013;143(5):648–55. 10.3945/jn.112.172668 23514765

[24] Garcia AL, Steiniger J, Reich SC, Weickert MO, Harsch I, Machowetz A, et al Arabinoxylan fibre consumption improved glucose metabolism, but did not affect serum adipokines in subjects with impaired glucose tolerance. Hormone and metabolic research = Hormon- und Stoffwechselforschung = Hormones et metabolisme. 2006;38(11):761–6. 10.1055/s-2006-955089 17111305

[25] Garcia AL, Otto B, Reich SC, Weickert MO, Steiniger J, Machowetz A, et al Arabinoxylan consumption decreases postprandial serum glucose, serum insulin and plasma total ghrelin response in subjects with impaired glucose tolerance. European journal of clinical nutrition. 2007;61(3):334–41. 10.1038/sj.ejcn.1602525 16988651

[26] Lu ZX, Walker KZ, Muir JG, O'Dea K. Arabinoxylan fibre improves metabolic control in people with Type II diabetes. European journal of clinical nutrition. 2004;58(4):621–8. 10.1038/sj.ejcn.1601857 15042130

[27] Alberti KG, Zimmet P, Shaw J, Group IDFETFC. The metabolic syndrome—a new worldwide definition. Lancet. 2005;366(9491):1059–62. S0140-6736(05)67402-8 [pii]. 16182882 10.1016/S0140-6736(05)67402-8

[28] Englyst HN, Kingman SM, Cummings JH. Classification and measurement of nutritionally important starch fractions. European journal of clinical nutrition. 1992;46 Suppl 2:S33–50. 1330528

[29] Suresh K. An overview of randomization techniques: An unbiased assessment of outcome in clinical research. Journal of human reproductive sciences. 2011;4(1):8–11. 10.4103/0974-1208.82352 21772732 PMC3136079

[30] Ingerslev AK, Theil PK, Hedemann MS, Laerke HN, Bach Knudsen KE. Resistant starch and arabinoxylan augment SCFA absorption, but affect postprandial glucose and insulin responses differently. The British journal of nutrition. 2014;111(9):1564–76. 10.1017/S0007114513004066 24507768

[31] Francis CY, Morris J, Whorwell PJ. The irritable bowel severity scoring system: a simple method of monitoring irritable bowel syndrome and its progress. Alimentary Pharmacology & Therapeutics. 1997;11(2):395–402. 9146781 10.1046/j.1365-2036.1997.142318000.x

[32] Svedlund J, Sjodin I, Dotevall G. GSRS—a clinical rating scale for gastrointestinal symptoms in patients with irritable bowel syndrome and peptic ulcer disease. Digestive diseases and sciences. 1988;33(2):129–34. 3123181 10.1007/BF01535722

[33] Lewis SJ, Heaton KW. Stool form scale as a useful guide to intestinal transit time. Scandinavian Journal of Gastroenterology. 1997;32(9):920–4. 10.3109/00365529709011203 9299672

[34] Stjernman H, Granno C, Jarnerot G, Ockander L, Tysk C, Blomberg B, et al Short health scale: a valid, reliable, and responsive instrument for subjective health assessment in Crohn's disease. Inflammatory bowel diseases. 2008;14(1):47–52. 10.1002/ibd.20255 17828783

[35] McCleary BV, Monaghan DA. Measurement of resistant starch. Journal of AOAC International. 2002;85(3):665–75. 12083259

[36] Bach Knudsen KE. Carbohydrate and lignin contents of plant materials used in animal feeding. Anim Feed Sci Tecnol. 1997;67(4):319–38. 10.1016/S0377-8401(97)00009-6

[37] Englyst HN, Cummings JH. Simplified method for the determination of total non-starch polysaccharides by gas-liquid chromatography of constituent sugars as alditol acetates. Analyst. 1984;109(7):937–42. 10.1039/AN9840900937 6283946

[38] Jensen MT, Cox RP, Jensen BB. Microbial production of skatole in the hind gut of pigs given different diets and its relation to skatole deposition in backfat. Animal Science. 1995;61(02):293–304. 10.1017/S1357729800013837

[39] Bokulich NA, Joseph CM, Allen G, Benson AK, Mills DA. Next-generation sequencing reveals significant bacterial diversity of botrytized wine. PloS one. 2012;7(5):e36357 10.1371/journal.pone.0036357 22563494 PMC3341366

[40] McInnis EA, Kalanetra KM, Mills DA, Maga EA. Analysis of raw goat milk microbiota: impact of stage of lactation and lysozyme on microbial diversity. Food Microbiology. 2015;46:121–31. 10.1016/j.fm.2014.07.021 25475275

[41] Caporaso JG, Kuczynski J, Stombaugh J, Bittinger K, Bushman FD, Costello EK, et al QIIME allows analysis of high-throughput community sequencing data. Nature methods. 2010;7(5):335–6. 10.1038/nmeth.f.303 20383131 PMC3156573

[42] DeSantis TZ, Hugenholtz P, Larsen N, Rojas M, Brodie EL, Keller K, et al Greengenes, a chimera-checked 16S rRNA gene database and workbench compatible with ARB. Applied and Environmental Microbiology. 2006;72(7):5069–72. 72/7/5069 [pii]. 16820507 10.1128/AEM.03006-05 PMC1489311

[43] Edgar RC. Search and clustering orders of magnitude faster than BLAST. Bioinformatics (Oxford, England). 2010;26(19):2460–1. 10.1093/bioinformatics/btq461 20709691

[44] Bokulich NA, Subramanian S, Faith JJ, Gevers D, Gordon JI, Knight R, et al Quality-filtering vastly improves diversity estimates from Illumina amplicon sequencing. Nature methods. 2013;10(1):57–9. 10.1038/nmeth.2276 23202435 PMC3531572

[45] Faith DP. Conservation evaluation and phylogenetic diversity. Biological Conservation. 1992;61(1):1–10. 10.1016/0006-3207(92)91201-3.

[46] Lozupone C, Knight R. UniFrac: a new phylogenetic method for comparing microbial communities. Applied and Environmental Microbiology. 2005;71(12):8228–35. 71/12/8228 [pii]. 16332807 10.1128/AEM.71.12.8228-8235.2005 PMC1317376

[47] Nordgaard I, Hove H, Clausen MR, Mortensen PB. Colonic production of butyrate in patients with previous colonic cancer during long-term treatment with dietary fibre (Plantago ovata seeds). Scandinavian Journal of Gastroenterology. 1996;31(10):1011–20. 8898423 10.3109/00365529609003122

[48] Schoenfeld DA. Statistical considerations for clinical trials and scintific experiments http://hedwig.mgh.harvard.edu/sample_size/size.html—cross2010 [cited 2015].

[49] Julious SA, Campbell MJ, Altman DG. Estimating sample sizes for continuous, binary, and ordinal outcomes in paired comparisons: practical hints. Journal of biopharmaceutical statistics. 1999;9(2):241–51. Epub 1999/06/24. 10.1081/bip-100101174 . 10379691

[50] Bland M. Cross-over trials https://www-users.york.ac.uk/~mb55/msc/trials/cross.htm2010 [cited 2015].

[51] Team RC. R: A Language and Environment for Statistical Computing. Vienna, Austria: R Foundation for Statistical Computing; 2013.

[52] Salonen A, Lahti L, Salojarvi J, Holtrop G, Korpela K, Duncan SH, et al Impact of diet and individual variation on intestinal microbiota composition and fermentation products in obese men. The ISME journal. 2014;8(11):2218–30. 10.1038/ismej.2014.63 24763370 PMC4992075

[53] Wu GD, Chen J, Hoffmann C, Bittinger K, Chen YY, Keilbaugh SA, et al Linking long-term dietary patterns with gut microbial enterotypes. Science (New York, NY). 2011;334(6052):105–8. 10.1126/science.1208344 PMC3368382 21885731

[54] Tojo R, Suarez A, Clemente MG, de los Reyes-Gavilan CG, Margolles A, Gueimonde M, et al Intestinal microbiota in health and disease: role of bifidobacteria in gut homeostasis. World J Gastroenterol. 2014;20(41):15163–76. Epub 2014/11/12. 10.3748/wjg.v20.i41.15163 ; PubMed Central PMCID: PMCPmc4223251. 25386066 PMC4223251

[55] Yang J, Keshavarzian A, Rose DJ. Impact of dietary fiber fermentation from cereal grains on metabolite production by the fecal microbiota from normal weight and obese individuals. Journal of medicinal food. 2013;16(9):862–7. 10.1089/jmf.2012.0292 24044495

[56] Macfarlane GT, Englyst HN. Starch utilization by the human large intestinal microflora. The Journal of applied bacteriology. 1986;60(3):195–201. 2423494 10.1111/j.1365-2672.1986.tb01073.x

[57] Margolles A, Sanchez B. Selection of a Bifidobacterium animalis subsp. lactis strain with a decreased ability to produce acetic acid. Applied and Environmental Microbiology. 2012;78(9):3338–42. 10.1128/AEM.00129-12 22389372 PMC3346482

[58] Fukuda S, Toh H, Taylor TD, Ohno H, Hattori M. Acetate-producing bifidobacteria protect the host from enteropathogenic infection via carbohydrate transporters. Gut microbes. 2012;3(5):449–54. 21214 [pii]. 22825494 10.4161/gmic.21214

[59] Tanner SA, Chassard C, Zihler Berner A, Lacroix C. Synergistic effects of Bifidobacterium thermophilum RBL67 and selected prebiotics on inhibition of Salmonella colonization in the swine proximal colon PolyFermS model. Gut pathogens. 2014;6(1):44-014-0044-y. eCollection 2014. 10.1186/s13099-014-0044-y PMC4215022 25364390

[60] Belenguer A, Duncan SH, Calder AG, Holtrop G, Louis P, Lobley GE, et al Two routes of metabolic cross-feeding between Bifidobacterium adolescentis and butyrate-producing anaerobes from the human gut. Applied and Environmental Microbiology. 2006;72(5):3593–9. 72/5/3593 [pii]. 16672507 10.1128/AEM.72.5.3593-3599.2006 PMC1472403

[61] Rios-Covian D, Gueimonde M, Duncan SH, Flint HJ, de Los Reyes-Gavilan CG. Enhanced butyrate formation by cross-feeding between Faecalibacterium prausnitzii and Bifidobacterium adolescentis. FEMS microbiology letters. 2015 Epub 2015/10/01. 10.1093/femsle/fnv176 . 26420851

[62] Duncan SH, Holtrop G, Lobley GE, Calder AG, Stewart CS, Flint HJ. Contribution of acetate to butyrate formation by human faecal bacteria. The British journal of nutrition. 2004;91(6):915–23. 10.1079/BJN20041150 15182395

[63] Bourriaud C, Robins RJ, Martin L, Kozlowski F, Tenailleau E, Cherbut C, et al Lactate is mainly fermented to butyrate by human intestinal microfloras but inter-individual variation is evident. Journal of applied microbiology. 2005;99(1):201–12. Epub 2005/06/18. 10.1111/j.1365-2672.2005.02605.x . 15960680

[64] Png CW, Linden SK, Gilshenan KS, Zoetendal EG, McSweeney CS, Sly LI, et al Mucolytic bacteria with increased prevalence in IBD mucosa augment in vitro utilization of mucin by other bacteria. The American Journal of Gastroenterology. 2010;105(11):2420–8. 10.1038/ajg.2010.281 20648002

[65] Belobrajdic DP, Bird AR, Conlon MA, Williams BA, Kang S, McSweeney CS, et al An arabinoxylan-rich fraction from wheat enhances caecal fermentation and protects colonocyte DNA against diet-induced damage in pigs. The British journal of nutrition. 2012;107(9):1274–82. 10.1017/S0007114511004338 22115395

[66] Poslusna K, Ruprich J, de Vries JH, Jakubikova M, van't Veer P. Misreporting of energy and micronutrient intake estimated by food records and 24 hour recalls, control and adjustment methods in practice. The British journal of nutrition. 2009;101 Suppl 2:S73–85. 10.1017/S0007114509990602 19594967

[67] Jonsdottir SE, Brader L, Gunnarsdottir I, Kally Magnusdottir O, Schwab U, Kolehmainen M, et al Adherence to the Nordic Nutrition Recommendations in a Nordic population with metabolic syndrome: high salt consumption and low dietary fibre intake (The SYSDIET study). Food & nutrition research. 2013;57: 10.3402/fnr.v57i0.21391 PMC3866840 24358036

[68] Hamer HM, Jonkers D, Venema K, Vanhoutvin S, Troost FJ, Brummer RJ. Review article: the role of butyrate on colonic function. Alimentary Pharmacology & Therapeutics. 2008;27(2):104–19. 10.1111/j.1365-2036.2007.03562.x 17973645

[69] Rigottier-Gois L, Bourhis AG, Gramet G, Rochet V, Dore J. Fluorescent hybridisation combined with flow cytometry and hybridisation of total RNA to analyse the composition of microbial communities in human faeces using 16S rRNA probes. FEMS microbiology ecology. 2003;43(2):237–45. 10.1111/j.1574-6941.2003.tb01063.x 19719684

[70] Kaneda T. Iso- and anteiso-fatty acids in bacteria: biosynthesis, function, and taxonomic significance. Microbiological reviews. 1991;55(2):288–302. 1886522 10.1128/mr.55.2.288-302.1991 PMC372815

[71] Cummings JH, Beatty ER, Kingman SM, Bingham SA, Englyst HN. Digestion and physiological properties of resistant starch in the human large bowel. The British journal of nutrition. 1996;75(5):733–47. 8695600 10.1079/bjn19960177

