# Supplementary material for: Effects of Arabinoxylan and Resistant Starch on Intestinal Microbiota and Short-Chain Fatty Acids in Subjects with Metabolic Syndrome: A Randomised Crossover Study
Source: PLoS One. 2016 Jul 19;11(7):e0159223. doi: 10.1371/journal.pone.0159223 (PMC4951149; doi:10.1371/journal.pone.0159223)
Supplement: S1 Information — English translation and the original version in Danish. (DOCX) [file pone.0159223.s001.docx]

| Trial protocol |
| --- |
| **ButCoIns – Metabolic and Immunologic Effects of Resistant Starch and Arabinoxylans in People with Metabolic Syndrome** |

[Content 1](#_Toc319306975)

Translated version of Protocol from 10.03.2012

[1.0 BaCKground 3](#_Toc319306976)

[2.0 AIM 4](#_Toc319306977)

[3.0 Hypothesis 4](#_Toc319306978)

[3.1 Metabolic hypothesis 4](#_Toc319306979)

[3.2 Gastrointestinal (GI) hypotheses 4](#_Toc319306980)

[4.0 trial participants 5](#_Toc319306981)

[4.1 Number 5](#_Toc319306982)

[4.2 inclusion criteria 5](#_Toc319306983)

[4.3 exclusion criteria 6](#_Toc319306984)

[5.0 Study design 6](#_Toc319306985)

[5.1 Experimental diet 8](#_Toc319306986)

[5.2 Summary of time schedule and visits 8](#_Toc319306987)

[5.3 Screening- visit 1 8](#_Toc319306989)

[5.4 Visit 2, 5, 8 og 11 9](#_Toc319306990)

[5.5 Visit 3, 6, 9 og 12 9](#_Toc319306991)

[5.6 Visit 4, 7, 10 og 13 9](#_Toc319306992)

[6.0 Endpoints 9](#_Toc319306993)

[6.1 Metabolic endpoint 9](#_Toc319306994)

[2.2 Gastroinstestinal endpoints 10](#_Toc319306995)

[7.0 METHODS AND HANDLING OF SAMPLE 10](#_Toc319306996)

[7.1 Blood samples 10](#_Toc319306997)

[7.2 Screening analyses 10](#_Toc319306998)

[7.3 Registration of dietary intake and questionnaires regarding intestinal function 10](#_Toc319306999)

[7.4 Blood samples og faecal samples 10](#_Toc319307000)

[7.5 Sigmoideoscopy and colonic biopsies 11](#_Toc319307001)

[7.6 Oral glucose tolerance test 11](#_Toc319307002)

[7.7 Diurnal blood pressure 11](#_Toc319307003)

[7.8 MR 12](#_Toc319307004)

[7.8.1 MRI: 12](#_Toc319307005)

[7.8.2 1H MRS: 12](#_Toc319307006)

[7.9 Meal test 12](#_Toc319307007)

[7.9.1 Test meal: 12](#_Toc319307008)

[7.9.2 Plasma analyses: 12](#_Toc319307009)

[7.9.3 Indirect calorimetry: 13](#_Toc319307010)

[7.9.4 Biopsies from adipose tissue: 13](#_Toc319307011)

[7.9.5 Urine collection: 13](#_Toc319307012)

[8.0 Recruitment 13](#_Toc319307013)

[9.0 risks 14](#_Toc319307014)

[10.0 Plublication 14](#_Toc319307015)

[11.0 Funding and economic conditions 15](#_Toc319307016)

[12.0 Research biobank 15](#_Toc319307017)

[12.1 Analyses in present project 15](#_Toc319307018)

[12.2 Materials in research biobank for furture studies 16](#_Toc319307019)

[13.0 Handling of Personal DATA 16](#_Toc319307020)

[14.0 Research Ethics STATEMENT 17](#_Toc319307021)

[15.0 References 18](#_Toc319307022)

**1.0 Background**

Lifestyle-related diseases are among the leading causes of premature death in Denmark. A sedentary lifestyle with almost constant access to easy degradable food with high energy and fat density and low in dietary fibre is considered one of the main reasons for this unfortunate development that has resulted in increased incidence of obesity, metabolic syndrome (MeS) and type 2 diabetes [1,2].

Metabolic Syndrome (MeS) is an umbrella term for a syndrome, which is characterized by abdominal obesity, hypertension, dyslipideamia, impaired glucose tolerance and insulin resistance. The incidence of MeS has increased dramatically in recent years and now appears in about 25% of the adult western population, where it increases the risk of type 2 diabetes and cardiovascular diseases[3,4].

The basic mechanisms by which diet is implicated in the pathogenesis of MeS, type 2 diabetes and cardiovascular disease, is poorly understood. Research illustrating the interaction of the physiological, cellular and genetic levels of dietary factors will provide an opportunity to shed light on the early stages in the pathogenesis of MeS. We will examine the effect of a diet in which the carbohydrate fraction has a more favourable composition on glucose and lipid metabolism as well as the gut immune function, compared to a standard Western diet (Western-style diet) which is less rich in dietary fibres. It could possibly lead to an individualized diet advice that can prevent the development of MeS and open to the development of new healthy foods. Arabinoxylan (AX) and resistant starch (RS) represent carbohydrates which may have a positive influence on insulin sensitivity, carbohydrate metabolism and lipid metabolism as well as colon immune function [5]. Dietary fibres are polymerized carbohydrates, which are resistant to small bowel digestion [6-8]. They are degraded in the colon, into short-chain fatty acids particularly butyrate. The short-chain fatty acids appear to play a central positive modulatory role in the metabolism and immune function in the intestine [9].

The composition of the diet has an impact on the production of butyrate in colon. More butyrate is produced in the proximal part of colon than in the distal part. This difference in the butyrate production may be linked to the higher propensity to develop disease (colon cancer, inflammatory bowel disease in the distal part of the colon compared with more proximal parts of colon). The reason is probably due to butyrate's ability to reduce oxidative stress and inflammatory mechanisms in the proximal part of colon compared with more distal parts [10]. Butyrate may inhibit nuclear factor kB (NF-kB) locally, and NF-kB plays a central role in the upregulated proinflammatory immune responses in inflammatory bowel disease [10,11]. A butyrate-enriched diet has resulted in increased insulin sensitivity in mice with metabolic syndrome (C57BL / 6J mouse) [12]. Beneficial effects on insulin sensitivity in humans have also been shown in response to resistant starch, in acute and long-term studies [13,14], and arabinoxylan after 5-6 weeks intervention[15,16].

A reduction of the postprandial triglyceride response is also seen after an arabinoxylan-rich diet

[16]. Elevated non-fasting triglyceride (TG > 2.2 mol/L) is an important independent predictor of cardiovascular disease. Especially the postprandial triglyceride response appears to be a sensitive predictor of increased CHD risk [17]. Our group has previously shown that elevated postprandial triglyceride response is an important risk marker for cardiovascular disease in type 2 diabetics [18].

There are other possible explanations for the positive impact of dietary fibres on carbohydrate metabolism. Thus, a dietary fibre increases the viscosity and thereby slows gastric emptying and reduces the absorption of food in the small intestine[6]. At the same time, the dietary fibres bind bile acids in the intestine, whereby the reabsorption is prevented improving the lipid profile [19].

The effect of a combination of arabinoxylan and resistant starch has not been previously tested. We want to clarify whether a diet rich in arabinoxylan and resistant starch, compared with a Western-style diet, has a positive effect on metabolic parameters and immune function in the gut of subjects with MeS.

# 2.0 Aim

The project aims to investigate whether four weeks of dietary intervention with a diet supplemented with AX and RS versus a Western-style diet has a positive effect on risk markers for Type 2 diabetes and cardiovascular disease in subjects with MeS. Concurrently, the objectives are to assess the impact of AX and RS on: inflammation markers, gene transcription in adipose tissue, intestinal cellular immune response and on the content of butyrate in the colon.

# 3.0 Hypotheses

## 3.1 Metabolic hypothesis

Increased intake of AX and RS has a positive effect on components of MeS i.e. reduces postprandial lipideamia, increases insulin sensitivity, improves glucose metabolism and reduces 24h blood pressure

## 3.2 Gastrointestinal hypotheses

1. Increased luminal levels of butyrate in the colon affect the mucosal immunity in the bowel and in the blood as measured by flow cytometric analyses of mononuclear cells and dendritic cells from intestinal biopsies as well as in peripheral mononuclear cells from blood
2. Increased butyrate in the colon lumen leads to a down regulation of T-cell activation in mucosa assessed by antigen stimulation (influenza virus, CD3, CD28 stimulation) of T cells isolated by flow cytometry from gut biopsies.
3. In vitro demonstration of the modified T-cell activation by butyrate stimulation of isolated immune cells in culture from both PBMC isolation and T cells acquired from biopsies.
4. Increased luminal concentration of butyrate leads to change in the mucosal immune function evaluated by the gene expression of 200 genes involved in immune function.
5. Microbiome changes in response to increased butyrate evaluated by 16S rRNA.

# 4.0 Participants

## 4.1 Number of participants

20 participants in a randomized crossover study estimated in 2 ways:

1. The number is based on power calculations previously made ​​with the postprandial triglyceride response as the primary endpoint. Statistical power of 80% and a detectable difference of 20% on average (α = 0.05) require a number of 10 subjects [20,21]. Because the studies are not strictly comparable and due to the risk of a significant dropout due to the extensive study program, we aim for recruitment of 20 subjects.
2. If the expected incensement in the concentration of faecal butyrate is used as an endpoint (and that the minimum expected and interesting increase is 50%, for practical reasons 1 x SD of mean butyrate concentrations in persons on Western-style diet) then the following power calculation applies:

There are few studies on faecal butyrate, which describes standard deviation of the mean value, but two studies allows for an estimate [22,23]. Based on SEM, SD and number of patients in these two studies, one can calculate the total number of participants in a crossover design using the program on this website: <http://hedwig.mgh.harvard.edu/sample_size/size.html#cross>

10 patients total of 80 % power and 5 % significant level

13 patients total of 90 % and 5 % significant level

## 4.2 Inclusion criteria

- Age 18-75 years
- Metabolic syndrome; minimum 3 of the 5 following risk factors [4]:
  - Caucasians with a waist ≥ 94 cm for men and ≥80 cm for women.
  - Fasting triglyceride ≥ 1,7 mmol/L (individuals on cholesterol lowering medication are included as long as they are in stable condition)
  - HDL < 1,03 mmol/L for men, < 1,29 mmol/L for women
  - Systolic blood pressure ≥ 130 mmHg and/or diastolic blood pressure ≥85 mm Hg or in stable antihypertensive treatment
  - Fasting p-glucose > 5,6 mmol/L.

## 4.3 Exclusion criteria

- Diabetes mellitus
- Gastrointestinal diseases:
  - Chronic inflammatory bowel disease
  - Microscopic (lymphocytic / collagen) colitis
  - Previous surgery on the small intestine or colon beyond appendectomy.
  - Bile salt malabsorption
  - Daily intake of NSAIDs in addition to low-dose aspirin
- Use of laxatives in the trial
- Implanted magnetisable metal and pacemaker (inhibits MRI)
- Waist circumference > 130 cm (inhibits MRI)
- Blood donors tapped within 3 months
- Anticoagulation (Marevan, Innohep®)
- Severe hepatic, renal or cardiovascular disease (clinical specialist assessment)
- B-haemoglobin < 8.1 mmol /L (men) or < 7.4 mmol / L (women)
- Plasma 25-OH-vitamin D 2 + D 3 <12.5 ng/L
- Dysregulated metabolic disorder
- Systemic corticosteroid therapy (prednisolone)
- Alcohol consumption over 21 units / week (men) or 14 units / week (women)
- Use of drugs
- Pregnancy or lactation
- Legal disqualification

5.0 Design

The experiment is part of a larger project called ButCoIns, which consists of three Work Packages (WP) with several subgroups. Research Professor Knud Erik Bach Knudsen, Department of Animal Science, Aarhus University (AU-HBS), is primarily responsible for the project.
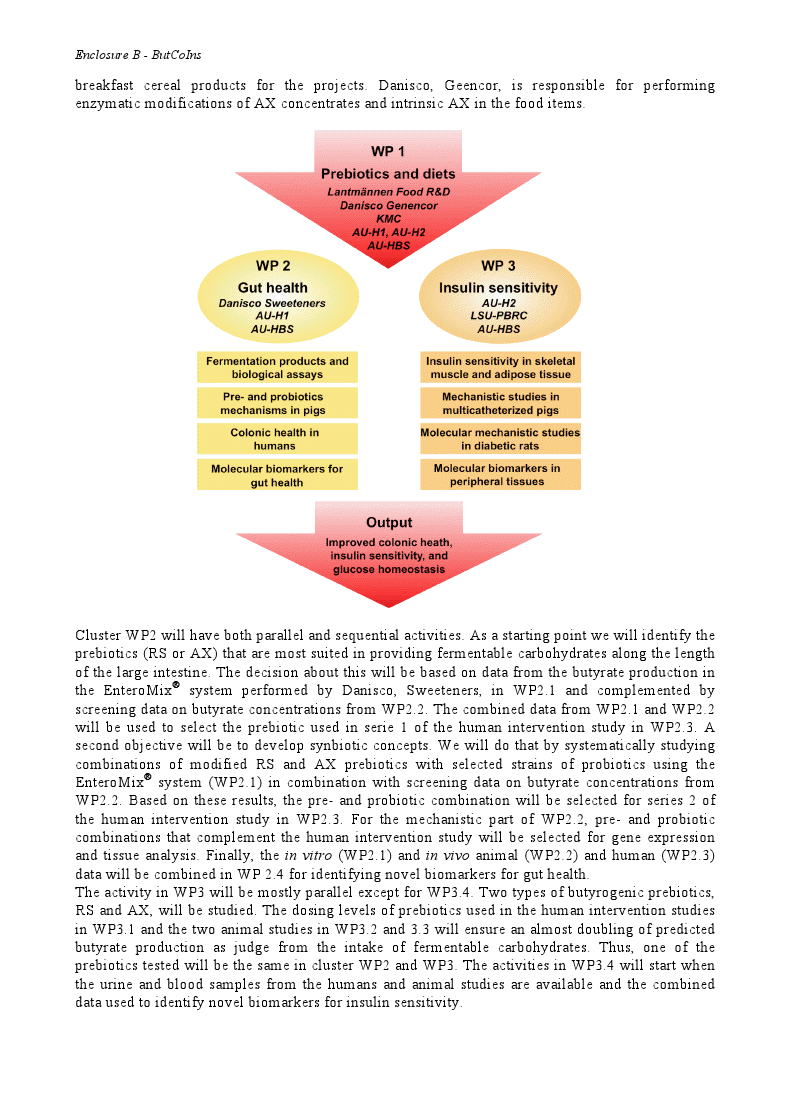


WP 1 is responsible for the production and the analyses of a variety of foods rich in AX and RS with the aim to increase colonic butyrate production. WP1 also produces food to the control diet, the Western-style diet.
The current project is carried out as a collaboration between WP3 (leader: professor MD, dr. med. Kjeld Hermansen and MD, Ph.D. Søren Gregersen AUH, Department of Endocrinology and Internal Medicine, (Dept. MEA), THG (AU-H2)) and WP2 (leader: professor, MD, dr. med. Hendrik Vilstrup and MD dr. med. Jens Dahlerup, AUH, Department of Hepatology and Gastroenterology, (dept. V), NBG (AU-H1)), Indtast tekst eller en webadresse til et website, eller [oversæt et dokument.](https://translate.google.dk/?tr=f&hl=da)

Oversæt fra: [Dansk](javascript:void(0))

focusing on the characteristics of MeS and colonic "health”.

MD Anne Grethe Schioldan, dept. MEA and MD Stine Hald, dept. V are responsible for the recruitment of subjects, study conduction (except for endoscopies which are performed by experienced physicians), and handling of diets and sample material. The study is a randomized single-blind crossover study with two diets of four weeks. 1: Healthy-carbohydrate diet (HCD) and 2: Western-style diet (WSD). A washout period of 4 to 8 weeks between the two intervention periods is planned. In the washout period the participants eat their habitual diet.

**10**

**Participants**

**4 weeks WSD**

**10**

**Participants**

**4 weeks HCD HCDHCDHCD**

**4 weeks WSD**

**4 weeks HCD**

**4 - 8 weeks washout period**

# 5.1 **Experimental diet**

**Healthy Carbohydrate Diet (HCD):** The subjects' usual diet is partly replaced by foods rich in resistant starch and arabinoxylan. Foods are supplied from Clinical Nutrition Research, dept. MEA, Aarhus University Hospital (AUH), Tage-Hansens Gade 2, 8000 Aarhus C.

**Western-style diet (WSD):** Four weeks in which the subject's usual diet is partially replaced by foods with a low content of AX and RS. Foods are supplied from Clinical Nutrition Research. Work Package 1 is responsible for the delivery of the intervention diets. Danisco Genencor, USA, is responsible for carrying out enzymatic modifications to AX from wheat bran and in cooperation with Lantmännen Food R & D incorporating them in foods like bread, pancakes and other products. Resistant starch is provided by KMC, Brande, Denmark. Lantmännen Food R & D supplies various milled flours, breads and cereals. Finally diet composition is based on calculations made by a clinical dietician from Clinical Nutrition Research, dept. MEA, AUH.

At visit 4 and 10, the subjects receive key food items and guidance from a dietician about how a part of the usual diet should be replaced. Food records (Appendix 7) are provided to the subjects in order to ensure compliance and detect any side effects. The diets aim to be isocaloric in order to keep the subjects’ body weight stable during the study. Dietary intake is recorded over three days during each diet intervention, and in the washout period.

## 5.2 Time Schedule and Visits

##

## 5.3 Screening- Visit 1

## The subjects meet fasting for the screening visit. Written informed consent is obtained. Blood samples are taken. Blood pressure and waist circumference are measured to make sure that the subjects fulfil the inclusion criteria. Medical history is obtained and the subjects are asked questions about alcohol habits, tobacco habits, diet and exercise. The screening decides if the subject can be included in the project. After inclusion, randomization to the order of the diets is performed. Questions about diet and bowel functions (bowel movements, abdominal pain, quality of life) are asked using standardized questionnaire (Appendix 7 and 8). The subjects are instructed not to make major lifestyle changes during the study because the study aims for stable body weight throughout the study.

## 5.4 Visit 2, 5, 8 and 11

## Sigmoideoscopy is performed before and after each four-week diet. The subjects meet at Department of Hepatology and Gastroenterology, AUH, NBG. The subjects are fasting and are asked to ensure that there has been bowel movement immediately before the endoscopy. If this is not possible, the subjects receive a clyster. Faeces samples and tissue biopsies from the intestine are obtained by an experienced physician (see below).

## 5.5 Visit 3, 6, 9 and 12

## OGTT, MR, diurnal blood pressure as well as questionnaire about bowel function are performed before and after each four-week diet. The subjects meet fasting to oral glucose tolerance test (OGTT) and application of blood pressure device at the Clinical Nutrition Research, Dept. MEA, AUH, THG. Subsequently, the subjects are transported to Skejby, where MRI is performed.

## 5.6 Visit 4, 7, 10 and 13

Meal Tolerance Test including Indirect Calorimetry is performed before and after each intervention period. Adipose tissue biopsies are taken at the end of the two intervention periods, i.e. visit 7 and 13. The subjects meet fasting at the Clinical Nutrition Research, Dept. MEA, AUH, THG. A predefined mixed meal is served. Indirect calorimetry is carried out before and twice after the ingestion of the test meal. Adipose tissue biopsies are taken before and after the meal. The meal tolerance test lasts approximately 6 hours and blood samples are taken at fixed time points. Urine is also being collected.

# 6.0 Endpoints

## 6.1 Metabolic Endpoints

1. Primary Metabolic Endpoint
   1. Postprandial triglyceride response[17]
2. Secondary Metabolic Endpoints
   1. Postprandial responses of p-glucose, p-insulin, p-C-peptide, p-incretins (GLP-1 and GIP), p-butyrate and p-short-chain fatty acids (SCFA) and Non Esterified Fatty Acid (NEFA) to the standardized mixed meal.
   2. Insulin sensitivity measured by Matsuda index[24], HOMA[25] and fructosamin.
   3. Triglyceride content in liver and muscle evaluated by MR spectroscopy[26,27]
   4. Gene expression in adipose tissue for the detection of differences between fasting and postprandial conditions.
   5. Energy consumption measured by indirect calorimetry.
   6. Changes in inflammatory markers in plasma (eg. hs CRP, IL-6 and adiponectin).
   7. Changes in endothelial biomarkers (eg. ICAM, VCAM).
   8. Diurnal blood pressure
3. 6.2 Primary Gastrointestinal Endpoints
   1. Mucosal T-cell activation expressed by reduced expression of CD25, CD56
   2. Changes in NF-κB, evaluated by gene expression analysis
   3. Changes in the production of proinflammatory cytokines at mucosal level (IFN-Υ, TNF-α, IL-17)
   4. Changes in the production of regulatory cytokines at mucosal level (IL-22, IL-10, TGF-β)
   5. Examine changes in cytokines in PBMC
4. Secondary Gastrointestinal Endpoints
   1. Faecal butyrate concentration before and after each intervention (Foulum)
   2. Modified phenotype of dendritic cells with increased expression of CD103.
   3. Modified expression of TNFR-II in response to increased butyrate concentration
   4. Modifications in Trefoil Factor measured in blood and at mucosal level (Holger Jon Møller, Dept. Clinical Biochemistry)
   5. Intestinal Health: Flatulence, number of bowel movements per day and quality of life.

# 7.0 Methods and handling of test material

## 7.1 Blood samples

## Blood samples are stored on ice and immediately centrifuged at 2000 G and 4^o^ C for 15 minutes. Plasma is frozen at -20^o^ C and subsequently stored at -80^o^ C.

## 7.2 Screening analyses

Blood samples are taken and analysed the same day for: Fasting p-glucose, p-lipids, HbA1c, p-Na^+^, p-K^+^, p-Creatinine, TSH, ALAT, CRP, leukocytes, albumin, 25-OH-D vitamin, PTH, alkaline phosphatase, haemoglobin, thrombocytes, prothrombin and APTT. Estimated blood volume: ***16 ml.*** The samples are analysed at Department of Clinical Biochemistry, Aarhus University Hospital (AUH). Debris is being destroyed. The samples can be taken up to 3 weeks prior to randomization.

7.3 Diet Registration and questionnaire on bowel function

Dietary intake is estimated by a clinical dietician using standardized food records completed before and during each four-week diet. The food record is based on the total dietary intake on two weekdays and one weekend day (Appendix 7). Together with the food record, a questionnaire based on *The Irritable bowel severity scoring system* [28]), *Gastrointestinal Symptoms Rating Scale* [29] *Bristol Stool Form Chart* [30] and *Short Health Scale* [31,32] must be filled in. The questionnaire is internally validated at dept. V in ten patients with irritable bowel syndrome or inflammatory bowel disease (Appendix 8).

## 7.4 Blood tests and faecal samples in the gastrointestinal part of the study

1. Subjects collect their faecal samples in disposable trays before endoscopy.

- 1. Faeces are analysed for calprotectin according to the department's practice.
  2. If stools are loose: Two faecal samples are cultured for detection of pathogenic intestinal bacteria and Clostridium difficile according to the department's practice.
  3. Faecal sample for 16S rRNA analysis of intestinal microbiota are frozen separately at -80°C.

d. Faecal samples for research biobank are frozen separately at -80°C.

## 7.5 Sigmoidoscopy and intestinal biopsies

Endoscopy with biopsies: experienced physicians from the research group at dept. V outpatient clinic perform sigmoidoscopy.

1. Biopsies:
   1. 2 x 2 from colon descendens and colon sigmoideum, respectively, are fixed in formalin and sent to pathological analyses for description and grading activity index according to established clinical practice.
   2. 2 x 8 biopsies are put into vials with RPMI and set up for the cell culture according to standard operation protocol [33,34] and for analysis by flow cytometry for activation markers and intracellular Th17 production.
   3. 2 x 5 biopsies are taken for detection of fibroblasts, dendritic cells and macrophages.
   4. 2 x 3 biopsies are taken for analyses of the expression of genes of the immune system and intestinal integrity.
   5. 2 x 3 biopsies are analysed for enzyme activity.

## A total of 42 biopsies are taken. Each biopsy corresponds to 1 / 20,000 of the total colonic surface area.

## 7.6 Oral glucose tolerance test

The subjects meet fasting, and a catheter is placed in the antecubital vein. They consume 75 g of D-glucose dissolved in 300 mL water within 5 minutes. Blood samples are taken from the peripheral venous catheter at the following time points: -15, -10, 0, 30, 60 and 120 minutes. Plasma is analysed for insulin and glucose in order to calculate insulin sensitivity using HOMA [25] and Matsuda index [24]. The analyses are performed in dept. MEA laboratory. Sixteen mL of blood is taken at each oral glucose tolerance test. Excess material will be destroyed.

Blood samples for Dept. V research are obtained from the same catheter. Here we take a total of 72 mL of blood (8 x 9 mL EDTA blood for whole blood flow cytometry and for the separation of blood mononuclear cells (PBMC), 1 x 9 ml of whole blood in a dry vial for serum and 1 x 9 ml EDTA blood plasma). PBMC, serum and plasma are prepared within 1 hour and frozen in the biobank at -140 ° C and -80 ° C, respectively.

Haemoglobin and 25-OH-vitamin D is checked at all crossovers and analysed by routine analysis of Clinical Biochemistry, AUH.

## 7.7 24h blood pressure

24h blood pressure device Spacelabs, (model 90207/90217, USA), is put on upon arrival at OGTT and removed 24h later.

## 7.8 MR

All MR acquisitions are obtained on MRI research centre AUH, Skejby. The scans take approximately for one hour.

7.8.1 MRI: T1-weighted whole body scan without contrast. Recording is performed lying with the arms elevated above the head and the scan goes from fingertips to toes [27].

## 7.8.2 1H MRS: MR proton spectroscopy is used to determine the fat content in the liver [27] and muscle [26]. The studies are supervised by Dr. Med. Hans Stødkilde-Jørgensen.

## 7.9 Meal tolerance test

7.9.1 Test meal:

The meal composition is the same for all four meal tests. The subjects encounter fasting (at least 8 hours) in the morning. They consume a mixed meal consisting of rye bread and white bread with butter, bacon, cheese, eggs and salami and a glass of low-fat milk and a cup of decaffeinated coffee or water. Total amount of energy 1043 kcal, with 75 g fat, 38 g protein and 49 g carbohydrates. The purpose of the high fat content of the meal (65%) is to achieve a significant postprandial lipid response. The meal should be consumed within 20 minutes from time 0. The analyses described below are performed at time 0 and during the subsequent 6 hours. To avoid elevated p-triglyceride prior to the meal, participants were instructed to avoid alcohol for two days prior to the meal tolerance test.

7.9.2 Plasma analyses:

| **Time (min)** | **0** | **15** | **30** | **60** | **120** | **180** | **240** | **360** |
| --- | --- | --- | --- | --- | --- | --- | --- | --- |
| Triglyceride | x |  |  | x | x | x | x | x |
| Glucose | x | x | x | x | x |  | x | x |
| Insulin | x | x | x | x | x |  | x | x |
| GLP-1 | x | x | x | x | x |  | x | x |
| GIP | x | x | x | x | x |  | x | x |
| ApoB-48 | x |  |  |  | x |  | x | x |
| Glucagon | x | x | x | x | x |  | x | x |
| SCFA incl. Butyrate | x |  |  | x | x | x | x | x |
| I-FABP | x |  |  |  |  |  |  |  |
| NEFA | x |  |  | x | x | x | x | x |
| Adiponectin | x |  |  |  |  |  |  |  |
| IL-6 | x |  |  |  |  |  |  |  |
| hsCRP | x |  |  |  |  |  |  |  |
| MCP-1 | x | x | x | x | x |  | x |  |
| Rantes/ CCL5 | x | x | x | x | x |  | x |  |
| PAI-1 | x |  |  |  |  |  |  |  |
| Fructosamin | x |  |  |  |  |  |  |  |
| vCAM, iCAM | x | x | x | x |  |  |  |  |
| iCAM | x | x | x | x |  |  |  |  |
| Metabolomics | x |  | x | x | x |  | x |  |

***All plasma analyses are made in Denmark;* Excess materials are being destroyed. *At each test meal approximately 150 mL whole blood are taken.***

7.9.3 Indirect calorimetry: The subject should lie completely still during the examination. After calibration of the calorimeter (Ventilated Hood, Deltatrac, Helsinki) a cloak of Plexiglas are placed over the subject's head. The cloak is fitted with tubes leading inhaled and exhaled air to Deltatrac. The device analyses CO2 release and O2 consumption, and thereby estimate the basal metabolic rate and the rate of glucose and lipid metabolism.

7.9.4 Biopsies from adipose tissue:

In local anaesthesia an incision in the abdominal skin (approximately 1 cm long) is made. Subcutaneous adipose tissue (about 5 g per biopsy) is taken using a Bergström biopsy. The adipose tissue is purified in isotonic buffered saline and frozen in liquid nitrogen. The skin is sutured and compressed. The biopsy is taken before and four hours after the start of the test meal. Genetic testing is performed using PCR technique at AROS Applied Biotechnology, Skejby.

Øverst på formularen

7.9.5 Urine collection: Urine is collected at the meal test for metabolomic analyses performed by our partners at Department of Animal Science, Aarhus University. The metabolomic analyses are done in order to get a metabolic fingerprint by 1 H-NMR and MS-based techniques [35]. 2 x 3 mL urine is taken from the six hours collection period. The samples are frozen and stored at -80° C. The rest of the urine is being discarded.

Øverst på formularen

# 8.0 Recruitment of subjects

Recruitment will be advertised in local newspapers and magazines, in the national database www.forsøgsperson.dk, and at local intranet, Aarhus University Hospital (Appendix 6). The ad states that subjects are wanted for a research project, and subjects who are interested in the project are invited to contact PhD student Anne Grethe Schioldan by phone or email. Written information (Appendix 2), the pamphlet "The subject's rights in a biomedical experiment" (Appendix 3) and a screening questionnaire (Appendix 4) are sent to them.
If the subject still want to enrol in the study appointments are made for information meetings. These meetings take place in small groups at the Clinical Nutrition Research, AUH, THG. At the information meetings purpose, background, aim, practical information, risks and possible complications are being described carefully. The subjects will be presented with a selection of the key food from the study. We will focus on that the subjects volunteer for the study and that they are free to withdraw from the study at any time. Subjects have the right to bring a companion to the information meetings. The meetings will take place in quiet surroundings led by one of the two PhD students, one of the endoscopists and a clinical dietician. Time is allocated at the meetings to answer any questions that the subjects may have.
If a subject decides to participate in the study an appointment is made for additional information in private. This will be pointed out at the information meeting. At the private information meeting the subjects also have the right to bring a companion. The subjects are informed about their right to refuse the knowledge of new essential health data that may be obtained during the study. Not until all the subject’s questions are answered a written a consent form (Appendix 5) is signed by the subject and the PhD student. Only when informed consent is obtained the screening (as described in details above) can begin in private.
If we obtain new knowledge about the risks and harms during the study the subjects will be informed orally and on paper. Essential new health information obtain during the study will be informed orally to the subject.
When the study is finished a summary of the results and conclusions will be send to the subjects.

# 9.0 Risks

- High-fibre diets can cause diarrhoea, increased flatulence and eructation.
- Blood sampling can cause bruises. A total of 1000 mL blood is taken over 13 to 16 weeks (Appendix 9). This corresponds with the donation of blood from a blood donor and can cause anaemia. At screening haemoglobin is measured in order to ensure that subject is not anaemic at enrolment. Blood donation within three months prior the enrolment is an exclusion criterion. In rare cases infection can occur at the injection site.
- Sigmoidoscopy, endoscopy of the distal 40 to 60 cm of the colon, is performed with a flexible scope (about 10 mm in diameter). Pain nerve fibres do not innervate the gut but endoscopy can cause mild to moderate abdominal discomfort. Several biopsies are taken during the endoscopy and there is a small risk of bleeding or perforation of the intestine, a risk that in elderly patients with sick bowel amounts to approximately 1 per 1,000 endoscopies [36]. In healthy subjects, the risk is assumed to be smaller. The risk is minimized by the fact that all endoscopies are made by the same two experienced endoscopists.
- MRI and indirect calorimetry can cause claustrophobia.
- Adipose tissue biopsies will create four small scars of 1-2 cm. Local anaesthetic can cause some tension and sting. The procedure may feel uncomfortably. There is a small risk of subsequent infection and bleeding and this is why the subjects will be advised against public swimming pools and hard physical activity a few days after the procedure. The subjects may experience a transient thickening or hardness in the area where the biopsy was taken. This may be due to bleeding or the formation of connective tissue.
- Participation in a study like this can cause unpredictable burdens for the subjects.

# 10.0 Publication

We aim to publish positive, negative and inconclusive results in international peer-reviewed journals and at conferences. The metabolic results will also be included in MD Anne Grethe Schioldans Ph.D. dissertation and gastrointestinal findings will be included in MD Stine Hald PhD thesis. A website will be made for the entire ButCoIns project and results will also be published here.

# 11.0 Funding and economic conditions

The study is part of a larger project called ButCoIns led by research professor Knud Erik Back Knudsen, Department of Animal Science, Aarhus University. Initiative to ButCoIns is taken by professor Kjeld Hermansen, professor Hendrik Vilstrup and research professor Knud Erik Bach Knudsen. The ph.d. thesis by MD, Anne Grethe Schioldan will be based on the endological part of the study where consultant Søren Gregersen is the principal supervisor and Professor Kjeld Hermansen is co-supervisor. The ph.d. thesis by MD, Stine Hald will be based on the gastrointestinal part of the study with Chief Physician, Dr. Med. Jens F. Dahlerup as principal supervisor and MD, ph.d. Jorgen Agnholt and MD, Anders Dige as co-supervisors. The gastrointestinal project also includes the professor, Dr. Med. Hendrik Willstrup, consultant, Dr. Med. Lisbet A. Christensen and Senior Medical Officer, ph.d. Christian Lodberg Hvas.

The trial is funded by the Research and Innovation board with 3,642,689 DDK for Clinical Nutrition Research MEA and 3,182,809 DDK to Medical Department V. Part of this funding will be allocated to salaries, experimental activities, operating costs and economic compensation to the subjects.
The compensation to subjects will be paid out in rates of 625 DDK per a) sigmoidoscopy (x4), b) glucose tolerance test including MRI and diurnal blood pressure (x4), c) meal test (x4) and d) adipose tissue biopsies (x4). In total taxable 10,000 DDK for completion of the study. Participation in the study can have a major impact on the subjects' daily Day. The intervention diet must be consumed as part of all main meals for two times four weeks, which for most subjects will require some planning. At the same time, subjects with non-storage space would have to pick up the diet intervention multiple times during the four weeks. Diet and faecal registration, which must be carried out over three days, four times during the trial, will also require a dedicated effort from the subjects. The study program includes as previously described endoscopy, adipose tissue samples, diurnal blood pressure and blood tests several times for each subject. The study can induce risks and side effects as previously described. Each subject must at least commit to 13 days to the study. The subject must be fasting, and prior to the meal test alcohol and tobacco must be avoid. The subjects should avoid strenuous activity for a few days after adipose tissue biopsies and refrain from public swimming pools until the wounds are healed. Subjects receive compensation for travel expenses and lost earnings according to the government rates. It should be noted that part of the test days take place on weekends and no lost earnings would be paid these days. The estimated mean age of the subjects’ is 50 to 60 years. Based on these considerations we find it reasonable to give our subjects this compensation. There are no pharmaceutical companies involved in this study. No one in the research group has private economic interests in the project.

**12.0 Research bio bank**

**12.1 Analyses in the present study**

The study materials are stored in established research bio banks in Medical Department V and Department of Endocrinology (MEA), AUH. The materials are kept locked in freezers at -80 ^o^ C. The samples are coded with subjects’ identification numbers, and these can only be linked to the individual subject by lists stored behind two locks at Medical Department V and MEA. The majority of the analyses will be conducted at AUH. Some of the materials will be analysed abroad (USA), and this will be written in the information materials to the subjects. Only materials for specific analyses will be send, and debris from analyses performed outside the Department of Medicine V or Department of Endocrinology will be destroyed.

Materials will be analyses at the following locations:

1. Department of Endocrinology, AUH
   1. Samples of adipose tissue: 5 g per biopsy
2. V-Research Laboratory, Department of Medicine V, AUH
   1. Faeces: 3 x 5-10 g faeces as "back-up" to further microbial characterization.
   2. Whole blood: Peripheral blood mononuclear cells, plasma, serum are analysed and frozen at -140° C and -80° C.
3. Department of Animal Science, Aarhus University (Appendix 11):
   1. Urine: 2 x 3 ml for metabolomics per meal test.
   2. Faecal samples: Two times 2-3 g per endoscopy for SCFA analysis and protein degradation.
   3. Plasma: 48 mL for metabolomics, SCFA and I-FABP
   4. Intestinal biopsies at endoscopy:
      1. 20 mg to qPCR is stored in 1.0 mL RNAlater. Biopsies are allowed to stand at room temperature for 24 hours, then stored at -80° C.
      2. 20 mg of enzyme activity frozen immediately in liquid nitrogen and stored at -80 ° C
4. Maria Marco, UC Davis, USA:

Faecal sample (5-10 g) for luminal microbiome analysis

**12.2 Materials in research bio bank for future projects**

In the study we will ask for permission (in the written consent (Appendix 5)) to store remaining materials after planned analyses in established research bio banks at Medical Department V and MEA, AUH. There will be no stored material outside the AUH. Use of materials from the research bio bank for future research will take place only after prior specific approval from the Research Ethics Committee.

# 13.0 Handling of personal data

All information on the subjects will be dealt with according to the law of the processing of personal data and the Danish Health act. All samples will be coded so only the investigators can connect samples to the individual subject. Test results are confidential and shall not be disclosed to third persons. The study will be reported to the Data Protection Agency and conducted in accordance with the Declaration of Helsinki.

# 14.0 Research ethics

The study will help to shed light on the potential beneficial effects of resistant starch and arabinoxylan on type 2 diabetes, cardiovascular disease and disease of the colon. The increasing prevalence of these diseases makes it desirable to find non-pharmacological approaches to prevent these conditions and to identify functional food products.
The experimentally prepared food will be made of plants approved for human nutrition. The products are manufactured under clean conditions and meet EFSA standards.
This study may benefits the subjects by giving them knowledge of their health status. Furthermore the subjects will receive a big part of their foods during the study. Financial compensation will be given to the subjects.
Overall, we think that the biomedical knowledge obtained in this study will be much more valuable than potential risks and harms of the subjects in this study.**15.0 References**

(1) Heidemann C, Schulze MB, Franco OH, et al. Dietary patterns and risk of mortality from cardiovascular disease, cancer, and all causes in a prospective cohort of women. Circulation 2008; 118: 230-7.

(2) Emberson JR, Whincup PH, Morris RW, et al. Lifestyle and cardiovascular disease in middle-aged British men: the effect of adjusting for within-person variation. Eur Heart J 2005; 26: 1774-82.

(3) Cornier MA, Dabelea D, Hernandez TL, et al. The metabolic syndrome. Endocr Rev 2008; 29: 777-822.

(4) Alberti KG, Eckel RH, Grundy SM, et al. Harmonizing the metabolic syndrome: a joint interim statement of the International Diabetes Federation Task Force on Epidemiology and Prevention; National Heart, Lung, and Blood Institute; American Heart Association; World Heart Federation; International Atherosclerosis Society; and International Association for the Study of Obesity. Circulation 2009; 120: 1640-5.

(5) Roberfroid M, Gibson GR, Hoyles L, et al. Prebiotic effects: metabolic and health benefits. Br J Nutr 2010; 104 Suppl 2: S1-63.

(6) Gemen R, De Vries PJF, Slavin JL. Relationship between molecular structure of cereal dietary fiber and health effects: Focus on glucose/insulin response and gut health. Nutr Rev 2011; 69: 22-33.

(7) Bach Knudsen KE, Serena A, Canibe N, et al. New insight into butyrate metabolism. Proc Nutr Soc 2003; 62: 81-6.

(8) McOrist AL, Miller RB, Bird AR, et al. Fecal butyrate levels vary widely among individuals but are usually increased by a diet high in resistant starch. J Nutr 2011; 141: 883-9.

(9) Denise Robertson M. Metabolic cross talk between the colon and the periphery: implications for insulin sensitivity. Proc Nutr Soc 2007; 66: 351-61.

(10) Hamer HM, Jonkers D, Venema K, et al. Review article: the role of butyrate on colonic function. Aliment Pharmacol Ther 2008; 27: 104-19.

(11) Canani RB, Costanzo MD, Leone L, et al. Potential beneficial effects of butyrate in intestinal and extraintestinal diseases. World J Gastroenterol 2011; 17: 1519-28.

(12) Gao Z, Yin J, Zhang J, et al. Butyrate improves insulin sensitivity and increases energy expenditure in mice. Diabetes 2009; 58: 1509-17.

(13) Robertson MD, Currie JM, Morgan LM, et al. Prior short-term consumption of resistant starch enhances postprandial insulin sensitivity in healthy subjects. Diabetologia 2003; 46: 659-65.

(14) Johnston KL, Thomas EL, Bell JD, et al. Resistant starch improves insulin sensitivity in metabolic syndrome. Diabet Med 2010; 27: 391-7.

(15) Lu ZX, Walker KZ, Muir JG, et al. Arabinoxylan fibre improves metabolic control in people with type II diabetes. Eur J Clin Nutr 2004; 58: 621-8.

(16) Garcia AL, Otto B, Reich SC, et al. Arabinoxylan consumption decreases postprandial serum glucose, serum insulin and plasma total ghrelin response in subjects with impaired glucose tolerance. Eur J Clin Nutr 2007; 61: 334-41.

(17) Kolovou GD, Mikhailidis DP, Kovar J, et al. Assessment and clinical relevance of non-fasting and postprandial triglycerides: an expert panel statement. Curr Vasc Pharmacol 2011; 9: 258-70.

(18) Carstensen M, Thomsen C, Gotzsche O, et al. Differential postprandial lipoprotein responses in type 2 diabetic men with and without clinical evidence of a former myocardial infarction. Rev Diabet Stud 2004; 1: 175-84.

(19) Dongowski G, Huth M, Gebhardt E. Steroids in the intestinal tract of rats are affected by dietary-fibre-rich barley-based diets. Br J Nutr 2003; 90: 895-906.

(20) Thomsen C, Rasmussen O, Lousen T, et al. Differential effects of saturated and monounsaturated fatty acids on postprandial lipemia and incretin responses in healthy subjects. Am J Clin Nutr 1999; 69: 1135-43.

(21) Thomsen C, Storm H, Holst JJ, et al. Differential effects of saturated and monounsaturated fats on postprandial lipemia and glucagon-like peptide 1 responses in patients with type 2 diabetes. Am J Clin Nutr 2003; 77: 605-11.

(22) Nordgaard I, Hove H, Clausen MR, et al. Colonic production of butyrate in patients with previous colonic cancer during long-term treatment with dietary fibre (Plantago ovata seeds). Scand J Gastroenterol 1996; 31: 1011-20.

(23) Beards E, Tuohy K, Gibson G. A human volunteer study to assess the impact of confectionery sweeteners on the gut microbiota composition. Br J Nutr 2010; 104: 701-8.

(24) Matsuda M, DeFronzo RA. Insulin sensitivity indices obtained from oral glucose tolerance testing: comparison with the euglycemic insulin clamp. Diabetes Care 1999; 22: 1462-70.

(25) Matthews DR, Hosker JP, Rudenski AS, et al. Homeostasis model assessment: insulin resistance and beta-cell function from fasting plasma glucose and insulin concentrations in man. Diabetologia 1985; 28: 412-9.

(26) Rico-Sanz J, Thomas EL, Jenkinson G, et al. Diversity in levels of intracellular total creatine and triglycerides in human skeletal muscles observed by (1)H-MRS. J Appl Physiol 1999; 87: 2068-72.

(27) Thomas EL, Hamilton G, Patel N, et al. Hepatic triglyceride content and its relation to body adiposity: a magnetic resonance imaging and proton magnetic resonance spectroscopy study. Gut 2005; 54: 122-7.

(28) Francis CY, Morris J, Whorwell PJ. The irritable bowel severity scoring system: a simple method of monitoring irritable bowel syndrome and its progress. Aliment Pharmacol Ther 1997; 11: 395-402.

(29) Svedlund J, Sjodin I, Dotevall G. GSRS--a clinical rating scale for gastrointestinal symptoms in patients with irritable bowel syndrome and peptic ulcer disease. Dig Dis Sci 1988; 33: 129-34.

(30) Lewis SJ, Heaton KW. Stool form scale as a useful guide to intestinal transit time. Scand J Gastroenterol 1997; 32: 920-4.

(31) Stjernman H, Granno C, Jarnerot G, et al. Short health scale: a valid, reliable, and responsive instrument for subjective health assessment in Crohn's disease. Inflamm Bowel Dis 2008; 14: 47-52.

(32) Hjortswang H, Jarnerot G, Curman B, et al. The Short Health Scale: a valid measure of subjective health in ulcerative colitis. Scand J Gastroenterol 2006; 41: 1196-203.

(33) Hvas CL, Kelsen J, Agnholt J, et al. Crohn's disease intestinal CD4+ T cells have impaired interleukin-10 production which is not restored by probiotic bacteria. Scand J Gastroenterol 2007; 42: 592-601.

(34) Agnholt J, Kaltoft K. In situ activated intestinal T cells expanded in vitro--without addition of antigen--produce IFN-gamma and IL-10 and preserve their function during growth. Exp Clin Immunogenet 2001; 18: 213-25.

(35) Bertram HC, Bach Knudsen KE, Serena A, et al. NMR-based metabonomic studies reveal changes in the biochemical profile of plasma and urine from pigs fed high-fibre rye bread. Br J Nutr 2006; 95: 955-62.

(36) Rabeneck L, Paszat LF, Hilsden RJ, et al. Bleeding and perforation after outpatient colonoscopy and their risk factors in usual clinical practice. Gastroenterology 2008; 135: 1899,1906, 1906.e1.

Appendix:

1. Lay summary
2. Participant information
3. Subjects’ rights when participating in biomedical studies
4. Screening questionnaire
5. Written consent
6. Ad for recruitment
7. Food record
8. Intestinal record
9. Volume calculation for blood sampling
10. Metal scheme
11. Biobank
12. Documentation for principal investigator
13. Principal investigator’s Curriculum Vitae

| Forsøgsprotokol |
| --- |
| **ButCoIns – Resistent stivelse og arabinoxylaners metaboliske og immunologiske effekter ved Metabolisk Syndrom** |

Indhold

10.03.2012

[Indhold 1](#_Toc319306975)

[1.0 Baggrund 3](#_Toc319306976)

[2.0 Formål 4](#_Toc319306977)

[3.0 Hypotese 4](#_Toc319306978)

[3.1 Metabolisk hypotese 4](#_Toc319306979)

[3.2 Gastrointestinale (GI) hypoteser 4](#_Toc319306980)

[4.0 Forsøgsdeltagere 5](#_Toc319306981)

[4.1 Antal 5](#_Toc319306982)

[4.2 Inklusionskriterier 5](#_Toc319306983)

[4.3 Eksklusionskriterier 6](#_Toc319306984)

[5.0 Studiedesign 6](#_Toc319306985)

[5.1 Forsøgsdiæten 8](#_Toc319306986)

[5.2 Oversigt over tidsperspektiv og antal besøg 8](#_Toc319306987)

[5.3 Screening- besøg 1 8](#_Toc319306989)

[5.4 Besøg 2, 5, 8 og 11 9](#_Toc319306990)

[5.5 Besøg 3, 6, 9 og 12 9](#_Toc319306991)

[5.6 Besøg 4, 7, 10 og 13 9](#_Toc319306992)

[6.0 Effektmål 9](#_Toc319306993)

[6.1 Metaboliske effektmål 9](#_Toc319306994)

[2.2 Gastroenterologiske (GI) effektmål 10](#_Toc319306995)

[7.0 Metoder og håndtering af prøvemateriale 10](#_Toc319306996)

[7.1 Blodprøver 10](#_Toc319306997)

[7.2 Screeningsanalyser 10](#_Toc319306998)

[7.3 Kostregistrering og spørgeskema om tarmfunktion 10](#_Toc319306999)

[7.4 GI-blodprøver og afføringsprøver 10](#_Toc319307000)

[7.5 Sigmoideoskopi og tarmbiopsier 11](#_Toc319307001)

[7.6 Oral glukose tolerance test 11](#_Toc319307002)

[7.7 Døgnblodtryk 11](#_Toc319307003)

[7.8 MR 12](#_Toc319307004)

[7.8.1 MRI: 12](#_Toc319307005)

[7.8.2 1H MRS: 12](#_Toc319307006)

[7.9 Måltidstest 12](#_Toc319307007)

[7.9.1 Testmåltidet: 12](#_Toc319307008)

[7.9.2 Plasmaanalyser: 12](#_Toc319307009)

[7.9.3 Indirekte kalorimetri: 13](#_Toc319307010)

[7.9.4 Fedtvævsbiopsier: 13](#_Toc319307011)

[7.9.5 Urinopsamling: 13](#_Toc319307012)

[8.0 Rekruttering af deltagere 13](#_Toc319307013)

[9.0 Risici forbundet med deltagelse 14](#_Toc319307014)

[10.0 Offentliggørelse 14](#_Toc319307015)

[11.0 Økonomiske forhold 15](#_Toc319307016)

[12.0 Forskningsbiobank 15](#_Toc319307017)

[12.1 Analyser i det konkrete projekt 15](#_Toc319307018)

[12.2 Materiale i forskningsbiobank til fremtidig forskning 16](#_Toc319307019)

[13.0 Behandling af personfølsomme data 16](#_Toc319307020)

[14.0 Videnskabsetisk redegørelse 17](#_Toc319307021)

[15.0 Referencer 18](#_Toc319307022)

# 1.0 Baggrund

Livsstilsrelaterede sygdomme er blandt de vigtigste årsager til tidlig død i Danmark. En stillesiddende livsstil med næsten konstant adgang til let nedbrydelige fødevarer med højt energiindhold og fedtdensitet samt lavt indhold af kostfibre betragtes som en af hovedårsagerne til denne uheldige udvikling, som har resulteret i øget forekomst af overvægt, metabolisk syndrom (MeS) og type 2 diabetes [1,2].

Metabolisk Syndrom (MeS) er en samlebetegnelse for et syndrom, som er karakteriseret ved abdominal fedme, forhøjet blodtryk, dyslipidæmi, nedsat glukosetolerans samt insulinresistens. Forekomsten af MeS er øget voldsomt de senere år og optræder nu hos ca. 25 % af den voksne vestlige befolkning, hvor den øger risikoen for type 2-diabetes og kardiovaskulær sygdom [3,4].

De basale mekanismer, hvorved kosten er involveret i patogenesen til MeS, type 2 diabetes og kardiovaskulær sygdom, er imidlertid dårligt belyst. Forskning, som belyser interaktionen på de fysiologiske, cellulære og genetiske niveauer af kostfaktorer, vil give mulighed for at belyse de tidlige stadier i patogenesen til MeS. Vi vil undersøge effekten af en kost, hvor kulhydratfraktionen har en mere gunstig sammensætning, på glukose- og lipidmetabolisme samt tarmens immunfunktion, sammenlignet med en almindelig vestlig kost (Western Style Diet), som er mindre rig på kostfibre. Det vil muligvis kunne lede til en individualiseret kostrådgivning, som kan forebygge udviklingen af MeS og åbne for udviklingen af nye sunde fødevarer.

Kostfibrene arabinoxylan (AX) og resistent stivelse (RS) repræsenterer kulhydrater, som muligvis har en positiv indflydelse på insulinfølsomhed, kulhydrat- og lipidmetabolismen samt tyktarmens immunfunktion[5]. Kostfibrene er polymeriserede kulhydrater, som er modstandsdygtige overfor tyndtarmens nedbrydning[6-8]. De nedbrydes i colon til blandt andet kortkædede fedtsyrer, herunder særligt butyrat. De kortkædede fedtsyrer ser ud til at spille en central positiv modulerende rolle for metabolisme og immunfunktion i tarmen[9].

Sammensætning af kosten har således betydning for den colons produktion af butyrat. Der produceres mere butyrat proximalt i tyktarmen end i den distale del. Denne forskel i butyratproduktionen kædes sammen med tilbøjeligheden til at udvikle sygdom fremfor alt distalt i tyktarmen (coloncancer, inflammatorisk tarmsygdom). Årsagen er formentlig betinget af butyrats evne til at reducere oxidativt stress, og begrænsning af inflammatoriske mekanismer proximalt i forhold til distalt i colon[10] Butyrat kan bl.a. inhibere NF-κB lokalt, og NF-κB har en central placering i det opregulerede proinflammatoriske immunrespons ved inflammatorisk tarmsygdom [10,11].

I forsøg på mus med metabolisk syndrom (C57BL/6J mus) har en butyrat-rig diæt resulteret i øget insulinfølsomhed[12]. Ligeledes har humane studier vist en gavnlig effekt på insulinfølsomheden efter indtagelse både af resistent stivelse i akutte og længerevarende studier [13,14] samt efter 5-6 ugers arabinoxylanholdig kost[15,16]. En reduktion af det postprandielle triglyceridrespons er desuden set efter arabinoxylanberiget kost[16]. Forhøjet ikke-fastende triglycerid (TG >2,2 mol/l) er en vigtig selvstændig prædiktor for kardiovaskulær sygdom. Især det postprandielle triglyceridrespons ser ud til at være en sensitiv prædiktor for øget IHS risiko [17]. Vores gruppe har således tidligere vist, at forhøjet postprandielt triglyceridrespons er en vigtig risikomarkør for kardiovaskulær sygdom ved type 2-diabetikere[18].

Der findes andre forklaringer på kostfibrenes mulige positive indflydelse på kulhydratmetabolismen. Således øger kostfibre bestående af arabinoxylaner fødens viskositet og forsinker derved mavesækkens tømning og mindsker optagelsen af føde i tyndtarmen[6]. Samtidig kan kostfibre binde galdesyrer i tarmen, hvorved reabsorptionen hindres og lipidprofilen bedres[19].

Effekten af kombinationen af arabinoxylaner og resistent stivelse, som begge øger produktionen af butyrat, er ikke tidligere testet. ***Vi ønsker at afklare om der, sammenlignet med en Western Style Diet, er en positiv effekt af en kost rig på arabinoxylaner og resistent stivelse målt på metaboliske parametre samt immunfunktionen i tarmen hos personer med MeS***.

# 2.0 Formål

Projektets formål er at undersøge, om 4 ugers kostintervention med en kost suppleret med AX og RS versus en Western Style Diet har positiv effekt på risikomarkører for Type 2-diabetes og kardiovaskulær sygdom hos personer med metabolisk syndrom. Samtidig er det formålet at vurdere effekten af Ax og RS på inflammationsmarkører samt gener i fedtvæv og på tarmens cellulære immunrespons samt på indholdet af butyrat i colon.

# 3.0 Hypotese

## 3.1 Metabolisk hypotese

Øget indtag af Arabinoxylaner (AX) og resistent stivelse (RS) i kosten har en positiv indflydelse på komponenterne ved MeS dvs. reducerer postprandiel lipæmi, øger insulinfølsomheden, forbedrer glukose metabolismen og reducerer døgnblodtrykket.

## 3.2 Gastrointestinale (GI) hypoteser

Øget luminalt indhold af butyrat i colon påvirker den mucosale immunitet i colon og i blodet målt ved flowcytometri af mononucleære celler og dendritceller fra tarmbiopsier samt i perifere blodmononucleære celler fra blod:

1. Ændringer i mucosal immunfunktion afspejles i perifert blod målt ved T-cellesammensætning og cytokinforhold (i absolut og relativ koncentration).
2. Øget butyrat i colon lumen medfører en nedregulering af T-celle aktivering i Mucosa bedømt ved antigen stimulation (influenzavirus, CD3, CD28 stimulation) af T-celler isoleret ved flowcytometri fra vævsprøver fra slimhinden.
3. In vitro demonstration af ændret T-celle aktivering ved butyratstimulation af isolerede immunceller i kultur både fra PBMC isolation og biopsi derivede T-celler.
4. Overordnet ændring i immunfunktion i Mucosa ved øget butyrat i lumen bedømt ved ændring af genexpression af 200 gener involveret i immunfunktion.
5. Ændret mikrobiom ved øget butyrat bedømt ved 16 S-RNA.

# 4.0 Forsøgsdeltagere

## 4.1 Antal

20 forsøgsdeltagere i et randomiseret overkrydsningsforsøg estimeret på 2 måder.

1) Antallet er baseret på styrkeberegning, der tidligere er foretaget mhp. det postprandielle triglyceridrespons som primært endepunkt. Statistisk styrke på 80 % og en detekterbar forskel på 20 % i gennemsnit (α = 0.05) kræver et antal på 10 forsøgspersoner [20,21]. Da forsøgene ikke er fuldstændig sammenlignelige og pga. af risiko for betydeligt frafald pga. det omfattende forsøgsprogram stiles mod rekruttering af 20 forsøgspersoner.

2) Hvis stigning i fæces koncentrationen af butyrat bruges som et end point (og at det den minimale forventede og interessante stigning er 50 % = for praktiske formål 1 x SD af mean butyrat koncentration hos personer på Western style diet) så gør følgende sig gældende mhp antalsberegning:

Der findes kun få undersøgelser vedrørende butyratkoncentrationen i fæces, som oplyser standarddeviation for mean værdi – men to undersøgelser giver mulighed for et estimat[22,23]. Hvis man går ud fra disse to undersøgelser, kan man ud fra SE og antal patienter beregne SD og hermed beregne total antal personer i et cross-over design ved anvendelse af program på denne hjemmeside:

<http://hedwig.mgh.harvard.edu/sample_size/size.html#cross>

10 patienter totalt med 80 % power og 5 % signifikansniveau

13 patienter totalt med 90 % og 5 % signifikansniveau.

Hvis der totalt inkluderer 20 p*ati*enter vil der være taget højde for et relativt stort skønnet frafald på op til 50 % af deltagerne samt den betydelige usikkerhed (pga. af brug af estimater) for antalsberegning udfra forventet stigning i butyratkoncentrationen i fæces.

Samlet vil total 20 personer (dvs. 2 grupper á 10 personer med overkrydsning mellem de to diæter) skulle deltage for at sikre at valide primære endepunktsdata opnås.

## 4.2 Inklusionskriterier

- Alder 18-75 år
- Metabolisk syndrom; minimum 3 af de 5 følgende risikofaktorer[4]:
  - Taljemål ≥ 94 cm for mænd, ≥80 cm for kvinder af dansk oprindelse (Dette fordi der gælder andre taljemål for andre etniciteter)
  - Faste-triglycerid ≥ 1,7 mmol/L (personer i kolesterolsænkende behandling inkluderes ved stabil medicindosering)
  - HDL < 1,03 mmol/L for mænd, < 1,29 mmol/L for kvinder
  - Systolisk blodtryk ≥ 130 mm Hg og/eller diastolisk blodtryk ≥85 mm Hg eller med kendt hypertension i stabil medicinsk behandling
  - Faste p-glukose > 5,6 mmol/L.

## 4.3 Eksklusionskriterier

- Diabetes mellitus
- Gastrointestinale sygdomme:
  - Kendt kronisk inflammatorisk tarmsygdom
  - Kendt mikroskopisk (lymfocytær/kollagen) colitis
  - Tidligere operativt indgreb på tyndtarm eller colon ud over appendiktomi.
  - Kendt galdesyremalabsorption
  - Dagligt indtag af NSAID-præparater ud over lavdosis acetylsalicylsyre
- Brug af laksantia i forsøgsperioden
- Indopereret magnetiserbart metal og pacemaker (forhindrer MR-scanning)
- Abdominalomfang >130 cm (forhindrer MR-scanning)
- Bloddonorer tappet indenfor de seneste 3 måneder eller personer der skal til planlagt blodtapning
- Antikoagulationsbehandling (Marevan, Innohep)
- Alvorlig lever-, nyre- eller hjertekarsygdom (klinisk speciallægevurdering)
- B-hæmoglobin <8,1 mmol/l (mænd) eller <7,4 mmol/l (kvinder)
- Plasma 25-OH-vitamin D2+D3 <12,5 ng/l
- Dysreguleret stofskiftelidelse
- Systemisk binyrebarkhormonbehandling (Prednisolon)
- Alkoholforbrug over 21 genstande/uge (mænd) eller 14 genstande/uge (kvinder)
- Brug af euforiserende stoffer
- Graviditet eller amning
- Juridisk inhabilitet

# 5.0 Studiedesign

Forsøget er en del af et større projekt kaldet ButCoIns, som består af 3 Work Packages (WP) med flere undergrupper. Forskningsprofessor Knud Erik Bach Knudsen fra Institut for Husdyrbiologi og -sundhed, Aarhus Universitet (AU-HBS), er hovedansvarlig for projektet.


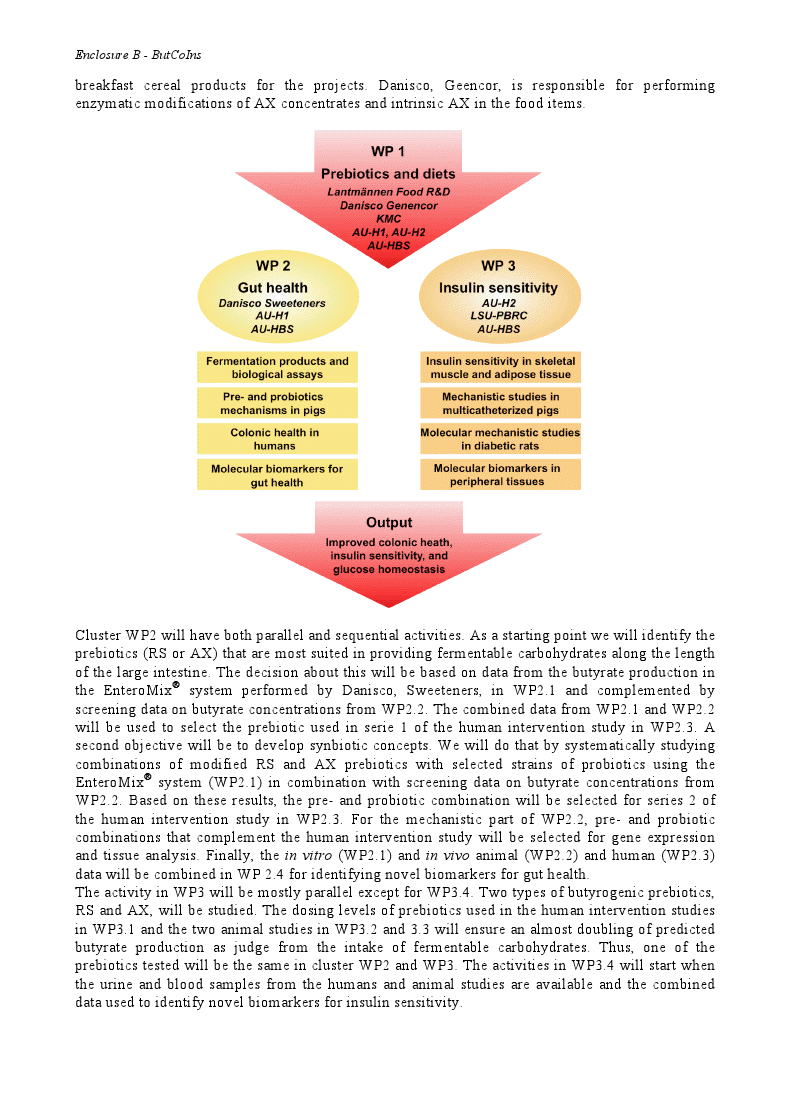


WP 1 forestår produktion og analyse af en række fødevarer med højt indhold af arabinoxylaner og resistent stivelse med henblik på at øge produktionen af butyrat i tarmen. Derudover produceres fødevarer til kontroldiæten Western Style Diet.

Projektet udføres som et samarbejde mellem WP3 (leder: professor overlæge, dr. med. Kjeld Hermansen samt overlæge, ph.d. Søren Gregersen AUH, MEA, THG (AU-H2)) og WP2 (leder: professor, overlæge, dr. med. Hendrik Vilstrup og overlæge dr. med. Jens Dahlerup, AUH, Medicinsk Hepato-gastroenterologisk afd. V, NBG (AU-H1)) med fokus på karakteristika ved MeS samt colon ”health”. Lægerne Anne Grethe Schioldan, afd. MEA, og Stine Hald, afd. V er ansvarlige for rekruttering af forsøgspersoner, udførelse af forsøg (fraset skopier, som udføres af erfarne skopører), og håndtering af diæter og prøvemateriale.

Studiet er et randomiseret enkeltblindet overkrydsningsforsøg. Hver forsøgsperson deltager i intervention med to diæter af fire ugers varighed, henholdsvis 1: Healthy Carbohydrate Diet (HCD) og 2: Western Style Diet (WSD). Imellem de to perioder planlægges 4-8 ugers udvaskningsperiode. Deltagerne spiser i udvaskningsperioden deres vanlige kost.

**10 deltagere:**

**4 ugers WSD**

**10 deltagere:**

**4 ugers HCD**

**4 ugers WSD**

**4 ugers HCD**

**4 - 8 ugers udvasknings-periode**

## 5.1 Forsøgsdiæten

**Healthy Carbohydrate Diet (HCD):** Forsøgspersonernes vanlige kost erstattes delvist af fødevarer beriget med resistent stivelse og arabinoxylaner udleveret fra Klinisk Ernæringsforskning, Aarhus universitetshospital (AUH), Tage-Hansens Gade 2, 8000 Aarhus C.

**Western Style Diet (WSD):** Fire uger, hvor forsøgspersonens vanlige kost delvist erstattes af fødevarer uden AX og RS udleveret fra Klinisk Ernæringsforskning. Work Package 1 er ansvarlig for levering af interventionskosten. Danisco Genencor, USA, er ansvarlig for at foretage enzymatiske modifikationer på AX fra hvedeklid og i samarbejde med Lantmännen Food R&D inkorporere dem i fødevarer som brød, pandekager og andre produkter. Resistent stivelse leveres af KMC, Brande, Danmark. Lantmännen Food R&D leverer forskellige formalede meltyper, brød og morgenmadsprodukter. Endelig diætsammensætning baseres på beregninger foretaget af en klinisk diætist fra Klinisk Ernæringsforskning, MEA, AUH.

Ved besøg 4 og 10 udleveres fødevarer, og forsøgspersonerne modtager vejledning af diætist om, hvordan en del af den vanlige kost erstattes. Der udleveres kostdagbøger (bilag 7), med henblik på at sikre compliance og registrere evt. bivirkninger. Diæterne tilstræbes isokaloriske, således at forsøgspersonerne holder stabil vægt under forsøget. Kosten registreres over tre dage under hver diæt, samt i udvaskningsperioden.

## 5.2 Oversigt over tidsperspektiv og antal besøg

## 5.3 Screening- besøg 1

Forsøgsdeltagerne møder fastende til screeningsbesøget. Her indhentes informeret samtykke til deltagelse i forsøget. Derefter tages blodprøver, måles blodtryk og taljemål for at sikre at forsøgspersonerne opfylder inklusionskriterierne. Der indhentes sygehistorie og spørges til alkohol- og tobaksvaner samt kost og motion. Screeningen danner baggrund for om forsøgspersonen kan indgå i projektet. Herefter randomiseres til rækkefølgen af diæterne.

Der spørges til kostvaner og vanlige afføringsforhold (afføringsfrekvens, mavesmerter, livskvalitet) ved hjælp af standardiseret spørgeskema (bilag 7 og 8). Forsøgsdeltagerne instrueres i ikke at foretage væsentlige livsstilsændringer i forbindelse med forsøget, idet stabil vægt tilstræbes under interventionsperioderne.

## 5.4 Besøg 2, 5, 8 og 11

Sigmoideoskopi før og efter hver 4-ugers diæt. Forsøgspersonerne møder på Medicinsk Afdeling V, AUH, NBG. De skal være fastende men ikke udrensede. Pt. anmodes om at kvittere/sikre, at der har været afføring umiddelbart inden skopien. Hvis dette ikke er muligt skal pt. udtømmes med fosfatklyx. Der tages fæces prøver og vævsbiopsier fra tarmen ved erfaren skopør (se nedenfor).

## 5.5 Besøg 3, 6, 9 og 12

OGTT, MR, døgnblodtryk samt spørgeskema om tarmfunktion før og efter hver 4-ugers diæt. Forsøgspersonerne møder fastende om morgenen på Klinisk Ernæringsforskning, AUH, THG til oral glukose tolerance test (OGTT) og påsætning af døgnblodtryksapparat. Efterfølgende transporteres forsøgspersonerne til Skejby, hvor der foretages MR-skanning.

## 5.6 Besøg 4, 7, 10 og 13

Måltidstest inklusiv Indirekte Kalorimetri udføres før og efter hver interventionsperiode. Fedtbiopsierne tages ved afslutning af de to interventionsperioder dvs. besøg 7 og 13. Forsøgspersonerne møder fastende på Klinisk Ernæringsforskning, AUH, THG. Forsøgspersonerne får serveret et forud defineret fedtrigt blandingsmåltid. Der foretages Indirekte Kalorimetri før og to gange efter indtagelse af testmåltidet. Fedtbiopsi tages før og efter måltidet. Måltidstesten varer ca. 6 timer og der tages løbende blodprøver og opsamles urin.

# 6.0 Effektmål

## 6.1 Metaboliske effektmål

1. Primære metaboliske effektmål
   1. Postprandielt triglyceridrespons[17]
2. Sekundære metaboliske effektmål
   1. Postprandielt respons efter blandingsmåltid (foruddefineret testmåltid) målt på p-glukose, p-insulin, p-C-peptid, p-incretiner (GLP-1 og GIP), p-butyrat og p-kort kædede fedtsyrer (SCFA), og Non Esterified Fatty Acid (NEFA).
   2. Insulinfølsomhed målt ved hjælp af Matsuda indeks[24], HOMA[25] og fructosamin.
   3. Fedtindhold i lever og muskel vurderet ved MR[26,27]
   4. Genekspression analyseret på fedtbiopsier. Med henblik på detektering af basale og postprandielle ændringer.
   5. Energiomsætning. Målt ved indirekte kaleometri ifm. måltidstest.
   6. Ændringer i inflammationsmarkører i plasma (f.eks. hs CRP, IL-6 og adiponectin).
   7. Ændringer i endothelcellebiomarkører (f.eks. ICAM, VCAM).
   8. Døgn BT

## 2.2 Gastroenterologiske (GI) effektmål

1. Primære GI effektmål
   1. T celle aktivering i mucosa udtrykt ved reduceret expression af CD25, CD56
   2. Ændring i NF-κB, bedømt ved genekspressionsanalyse
   3. Ændring i produktion af proinflammatoriske cytokiner på mucosalt niveau (IFN-Υ, TNF-α, IL-17)
   4. Ændring i produktion af regulatoriske cytokiner på mucosalt niveau (IL-22, IL-10, TGF-β)
   5. Undersøge for samme cytokinskifte i PBMC
2. Sekundære GI effektmål
   1. Måling af butyrat koncentrationen i fæces før og efter (Foulum)
   2. Ændret fænotype i dendritiske celler med øget expression af CD103.
   3. Ændret expression af TNFR-II ved øget butyrat
   4. Ændringer i Trefoil Factor mhp. anticarcinogene effekt – målt på blod og mucosalt niveau (Holger Jon Møller, Klinisk Biokemisk Afdeling)
   5. Tarmsundhed: Luft i maven, antal afføringer pr. dag, ildelugtende flatus samt livskvalitet.

# 7.0 Metoder og håndtering af prøvemateriale

## 7.1 Blodprøver

Efter blodprøvetagning opbevares prøverne på is, indtil de hurtigst muligt centrifugeres ved 2000 G og 4^o^ C i 15 minutter. Plasma nedfryses til -20^o^ C og opbevares efterfølgende ved -80^o^ C.

## 7.2 Screeningsanalyser

Blodprøve tages og analyseres samme dag for: Faste p-glukose, p-lipider, HbA1c, p-Na^+^, p-K^+^, p-Creatinin, TSH, ALAT, C-reaktivt protein, leukocytter, albumin, 25-OH-D vitamin, PTH, basisk phosphatase, Hgb, trombocyttal, protrombin og APTT. ***Blodvolumen ca. 16 ml. Prøverne analyseres på Klinisk biokemisk laboratorium, Aarhus Universitetshospital (AUH); restmateriale destrueres. Prøverne kan tages optil 3 uger før randomisering.***

## 7.3 Kostregistrering og spørgeskema om tarmfunktion

Kostregistrering foretages af klinisk diætist ved standardiseret kostanamnese før og under hver 4-ugers diæt samt i udvaskningsperioden. Kostanamnesen baseres på det totale kostindtag på to hverdage og en weekenddag (bilag 7).

Samtidig besvares et standardiseret spørgeskema om tarmfunktion er baseret på *The Irritable bowel severity scoring system* [28]), *Gastrointestinal Symptoms Rating Scale*[29] *Bristol Stool Form Chart* [30] og *Short Health Scale* [31,32]. Spørgeskemaet er internt valideret på Medicinsk Afdeling V på 10 patienter med irritabel tyktarm eller inflammatorisk tarmsygdom (bilag 8).

## 7.4 GI-blodprøver og afføringsprøver

1. Afføringsprøver indsamles ved, at patienten på engangsbakke opsamler afføringsprøve fra første afføring på undersøgelsesdagen eller fra afføring umiddelbart før sigmoideoskopien.
   1. Afføring til måling af calprotectin efter afdelingens vanlige praksis.
   2. Ved tynd afføring: To afføringsprøver til dyrkning for tarmpatogene bakterier og *Clostridium difficile* efter afdelingens vanlige praksis.
   3. Afføringsprøve til 16S-rRNA analyse for intestinal mikrobiota (snapfrysning separat ved -80 °C).
   4. Afføringsprøve til forskningsbiobank (snapfrysning separat ved -80 °C).

## 7.5 Sigmoideoskopi og tarmbiopsier

Endoskopi med biopsier: Sigmoideoskopi udføres i V-dagafsnit af erfaren endoskopør, der er læge fra projektgruppen. Med hensyn til mulig forberedelse – se ovenfor.

1. Biopsier:
   1. 2 x 2 fra hhv. colon descendens og colon sigmoideum fikseres i formalin og sendes til patologisk-anatomisk beskrivelse og graduering af aktivitetsindeks efter etableret klinisk praksis.
   2. 2 x 8 biopsier til spidsglas med RPMI og opsættes dels til celledyrkning efter laboratoriets standardprotoko[33,34] og dels til analyse med flowcytometri for aktiveringsmarkører og intracellulær Th17-produktion.
   3. 2 x 5 biopsier til undersøgelse for fibroblaster og dendritiske celler og makrofager.
   4. 2 x 3 biopsier til analyser for ekspression af gener i immunapparatet og intestinal integritet.
   5. 2 x 3 biopsier til analyse af enzymaktiviteten.

I alt tages 42 biopsier, svarende til samlet 1/20.000 af den samlede tyktarmsoverflade.

## 7.6 Oral glukose tolerance test

Forsøgsdeltagerne møder fastende og får anlagt perifert venekateter i fossa cubiti. De indtager 75 g D-glukose opløst i 300 ml vand indenfor maksimalt 5 minutter. Der tages blodprøver fra det perifere venekateter til tiderne: -15,-10, 0, 30, 60 og 120 minutter. Plasma analyseres for insulin og glukose med henblik på beregning af insulinfølsomhed ved hjælp af HOMA[25] og Matsuda-indeks[24]. ***Analyserne foretages i eget laboratorium. Der udtages ca. 16 ml blod per OGTT; restmateriale destrueres.***

***Blodprøver til afd V-forskning udtages ved samme indstik, i alt 72 ml: 8 x 9 ml EDTA-blod til fuldblodsflowcytometri og til separation af blodmononucleære celler (PBMC), 1 x 9 ml fuldblod på tørglas til serum og 1 x 9 ml EDTA-blod til plasma. PBMC, serum og plasma separeres inden for 1 time og nedfryses i forskningsbiobank ved hhv. -140°C og -80°C.***

***Hæmoglobin og 25-OH-D vitamin kontrolleres ved alle overgange og analyseres ved rutine analyser på Klinisk Biokemisk Afdeling, AUH.***

## 7.7 Døgnblodtryk

Døgnblodtryksapparat Spacelabs, (model 90207/90217, USA), bliver monteret ved ankomst til OGTT og afmonteres et døgn senere.

## 7.8 MR

Alle MR-optagelser foregår på MR-research Center AUH, Skejby. Skanningerne tager ca. en time per forsøgsperson.

7.8.1 MRI: T_1_-vægtet helkrops skanning uden kontrast. Optagelsen foretages liggende med armene strakt over hovedet og skanningen går fra fingerspidser til tæer.[27]

7.8.2 1H MRS: MR protonspektroskopi bruges til bestemmelse af fedtindholdet i leveren[27] og muskulaturen[26]. Undersøgelserne superviseres af dr. med. Hans Stødkilde-Jørgensen.

## 7.9 Måltidstest

7.9.1 Testmåltidet: Måltiderne er ens for alle fire måltidstests. Forsøgspersonerne møder fastende (minimum 8 timer) om morgenen. De indtager et blandingsmåltid bestående af rugbrød og hvidt brød med smør, bacon, ost, æg og salami samt et glas letmælk og en kop koffeinfri kaffe eller vand. Samlet energimængde 1.043 kcal, som fordeler sig på 75 g fedt, 38 g protein og 49 g kulhydrater. Formålet med måltidets høje fedtprocent (65 %) er at opnå et signifikant postprandielt lipidrespons. Måltidet skal indtages indenfor 20 minutter fra tiden 0, og forsøgspersonerne gennemgår de nedenfor beskrevne undersøgelser ved baseline og i løbet af de efterfølgende 6 timer. For at undgå forhøjet p-triglycerid forud for måltidet instrueres forsøgspersonerne i at undgå indtagelse af alkohol i to døgn op til måltidstesten.

7.9.2 Plasmaanalyser:

| **Tid (min)** | **0** | **15** | **30** | **60** | **120** | **180** | **240** | **360** |
| --- | --- | --- | --- | --- | --- | --- | --- | --- |
| Triglycerid | x |  |  | x | x | x | x | x |
| Glukose | x | x | x | x | x |  | x | x |
| Insulin | x | x | x | x | x |  | x | x |
| GLP-1 | x | x | x | x | x |  | x | x |
| GIP | x | x | x | x | x |  | x | x |
| ApoB-48 | x |  |  |  | x |  | x | x |
| Glukagon | x | x | x | x | x |  | x | x |
| SCFA inkl. Butyrat | x |  |  | x | x | x | x | x |
| I-FABP | x |  |  |  |  |  |  |  |
| NEFA | x |  |  | x | x | x | x | x |
| Adiponectin | x |  |  |  |  |  |  |  |
| IL-6 | x |  |  |  |  |  |  |  |
| hsCRP | x |  |  |  |  |  |  |  |
| MCP-1 | x | x | x | x | x |  | x |  |
| Rantes/ CCL5 | x | x | x | x | x |  | x |  |
| PAI-1 | x |  |  |  |  |  |  |  |
| Fructosamin | x |  |  |  |  |  |  |  |
| vCAM, iCAM | x | x | x | x |  |  |  |  |
| iCAM | x | x | x | x |  |  |  |  |
| Metabolomics | x |  | x | x | x |  | x |  |

***Alle plasmaanalyser foretages i Danmark; restmateriale destrueres. Ved hver måltidstest tages ca. 150 ml fuldblod.***

7.9.3 Indirekte kalorimetri: Forsøgspersonen skal ligge helt stille ved undersøgelsen. Efter kalibrering af kalorimetret (Ventilated Hood, Deltatrac, Helsinki) placeres en kappe af plexiglas over forsøgspersonens hoved. Kappen er forsynet med slanger som leder ind- og udåndingsluft til Deltatrac. Apparatet analyserer CO_2_-frigivelse og O_2_- forbrug, hvorved basal metabolisk rate samt glukose- og lipidmetabolisk rate kan estimeres.

7.9.4 Fedtvævsbiopsier: Efter anlæggelse af lokalanæstesi gøres 1 cm incision i abdominalhuden. Der udtages ca. 5 g. subkutant fedtvæv per biopsi ved hjælp af en Bergström biopsinål. Fedtvævet renses ved hjælp af isotonisk saltvand og fryses derefter i flydende kvælstof. Huden lukkes med et sting og der anlægges kompression. Biopsi foretages før og fire timer efter start på testmåltidet. ***Der foretages genanalyser ved hjælp af PCR-teknik på AROS Applied Biotechnology, Skejby.***

7.9.5 Urinopsamling: Der foretages urinopsamling i forbindelse med måltidstesten mhp. metabolomisk analyse hos vores samarbejdspartnere på Institut for Husdyrbiologi og – sundhed, Aarhus Universitet. Dette gøres med henblik på analyse af det metaboliske fingeraftryk ved hjælp af 1H-NMR- og MS-baserede teknikker[35]. Fra urinopsamlingen, som løber over 6 timer, udtages 2 prøver á 3 ml. Prøverne nedfryses og opbevares ved -80^o^ C. ***Resten af urinen kasseres.***

# 8.0 Rekruttering af deltagere

Rekruttering foregår via annoncering i lokale aviser og blade, samt via den nationale database [www.forsøgsperson.dk](http://www.forsøgsperson.dk) og Aarhus Universitetshospitals lokale intranet (bilag 6). I annoncen fremgår det, at der er tale om hvervning af forsøgspersoner til et forskningsprojekt. Interesserede opfordres til at kontakte ph.d.-studerende Anne Grethe Schioldan via telefon eller e-mail. Ved henvendelse tilsendes skriftlig deltagerinformation (bilag 2), pjecen ”Forsøgspersonens rettigheder i et biomedicinsk forsøg” (bilag 3) og et screeningsspørgeskema (bilag 4).

Såfremt personen efter gennemlæsning af det tilsendte materiale stadig er interesseret i at deltage i forsøget, aftales tid til et informationsmøde, som foregår i mindre grupper på Klinisk Ernæringsforskning, AUH, THG. Ved informationsmødet informeres grundigt om formål, baggrund og praktisk udførelse af forsøget, herunder risici og mulige komplikationer. Forsøgspersonerne vil blive præsenteret for et udvalg af de testfødevarer, som skal indtages under studiet. Der bliver endvidere lagt vægt på, at deltagelse er fuldstændig frivillig, og at et eventuelt ønske om at trække sig fra studiet til enhver tid vil blive accepteret, uden at forsøgspersonen skal begrunde dette. Der er ved informationsmødet mulighed for at medbringe en bisidder. Mødet vil foregå i rolige omgivelser ledet af én af de to ph.d.-studerende, en skopør samt en klinisk diætist. Der vil være afsat tid til at besvare eventuelle spørgsmål fra deltagerne.

***Hvis forsøgspersonen beslutter sig til eventuelt at deltage, aftales tid til information i enerum givet af en af de to ph.d.-studerende. Dette gøres der opmærksom på ved informationsmødet. Her er der også mulighed for bisidder. Forsøgsdeltageren informeres om retten til at frabede sig viden om nye væsentlige helbredsoplysninger, som måtte fremkomme under forsøget. Når eventuelle spørgsmål er besvaret, underskriver forsøgsdeltageren en samtykkeerklæring (bilag 5) til deltagelse i forsøget. Først når informeret samtykke er indhentet begynder screeningsundersøgelse i enrum - som beskrevet under tidligere afsnit.***

Hvis der under forsøget fremkommer ny viden om ulemper og risici, eller der, efter godkendelse fra Videnskabsetisk Komité, foretages ændringer i forsøgsgangene, bliver forsøgsdeltagerne informeret mundtligt og skriftligt om dette. Hvis der fremkommer væsentlige nye helbredsoplysninger om den enkelte forsøgsperson, vil vedkommende blive informeret mundtligt om dette.

Når forsøget er afsluttet, får alle forsøgsdeltagere tilsendt et lægmandsresume af forsøgets resultater og konklusioner.

# 9.0 Risici forbundet med deltagelse

- Fiberrig kost kan i nogle tilfælde give anledning til diaré, øget flatulens og bøvsetendens.
- Blodprøvetagning kan give blåt mærke i albuebøjningen. Der udtages i alt ca. 1000 ml i de 13-16 uger (Bilag 9)forsøget strækker sig over. Dette svarer til tapning som bloddonor, men indebærer risiko for blodmangel (anæmi). Ved screeningen tages hæmoglobin for at sikre, at forsøgspersonen ikke har forudgående anæmi, og personer, som har doneret blod indenfor de seneste 3 måneder, ekskluderes. ***Der kan i sjældne tilfælde opstå infektion i tilknytning til indstiksstedet.***
- Ved sigmoideoskopi, der er en kikkertundersøgelse af de nederste 40-60 cm af tyktarmen, anvendes en bøjelig kikkert (slange) som er ca. 10 mm i diameter. Man har ikke smertetråde i tarmen, så derfor opleves fremførelsen af kikkerten som en let til moderat pressende fornemmelse som ved luft i maven. Der tages en række vævsprøver, som man ikke kan mærke. Ved vævsprøvetagning er der en lille risiko for blødning eller hul på tarmen, en risiko som hos ældre patienter med syg tarm er opgjort til ca. 1 pr 1000 undersøgelser [36]. Hos raske kan risikoen antages at være mindre. Risikoen minimeres yderligere af, at alle skopier udføres af samme to erfarne skopører.
- MR skanning og indirekte kalorimetri kan give klaustrofobi.
- Fedtbiopsierne vil give anledning til 4 små ar, der er dog tale om små incisioner på 1-2 cm. Anlæggelse af lokalbedøvelse kan spænde og svie lidt, og selve biopsitagningen kan føles lidt ubehagelig, men giver ikke smerte. Der er en lille risiko for efterfølgende infektion og blødning, ***hvorfor svømmehalsbesøg og hård fysisk aktivitet frarådes nogle dage efter indgrebet. Forsøgspersonerne kan opleve en forbigående fortykkelse eller hårdhed i området, hvor biopsien er taget. Dette kan skyldes blødning eller bindevævsdannelse og fortager sig over tid.***
- Der kan opstå uforudsigelige belastninger i forbindelse med deltagelse i et sundhedsvidenskabeligt forsøg.

# 10.0 Offentliggørelse

Målet er at offentliggøre både positive, negative *og inkonklusive* resultater i internationale peer-review tidsskrifter og ved konferencer. De metaboliske resultater vil endvidere indgå i cand. med. Anne Grethe Schioldans ph.d. afhandling mens de gastrointestinale resultater vil indgå i cand. med. Stine Halds ph.d. afhandling. I forbindelse med projektet vil der endvidere blive oprettet en samlet hjemmeside for ButCoIns, hvor resultater vil blive offentliggjort.

# 11.0 Økonomiske forhold

Forsøget er en del af et større projekt kaldet ButCoIns, som ledes af forskningsprofessor Knud Erik Back Knudsen, Institut for Husdyrbiologi og –sundhed, Aarhus Universitet. Initiativ til forsøget er taget af professor Kjeld Hermansen, professor Hendrik Vilstrup, og forskningsprofessor Knud Erik Bach Knudsen. Den endokrinologiske del af forsøget skal ligge til grund for Cand. Med. Anne Grethe Schioldans Ph.d. afhandling, hvor overlæge Søren Gregersen er hovedvejleder og professor Kjeld Hermansen er medvejleder. Den gastrointestinale del af forsøget skal ligge til grund for cand. med. Stine Halds ph.d. afhandling, hvor overlæge, dr. med. Jens F. Dahlerup er hovedvejleder og overlæge, ph.d. Jørgen Agnholt samt cand. med. Anders Dige er medvejledere. Den gastrointestinale projektgruppe består herudover af professor, dr. med Hendrik Vilstrup, overlæge, dr. med. Lisbet A. Christensen samt afdelingslæge, ph.d. Christian Lodberg Hvas.

Forsøget er finansieret via Forsknings- og innovationsstyrelsen med 3.642.689 kroner til Klinisk Ernæringsforskning MEA og 3.182.809 kroner til Medicinsk Afdeling V. Støtten indgår i projektet til løn, forsøgsaktiviteter, driftsudgifter og økonomisk ulempegodtgørelse til forsøgsdeltagerne.

***Den skattepligtige ulempegodtgørelse til forsøgspersonerne udbetales i rater på 625 kroner per a) Sigmoideoskopi (x4), b) glukosebelastningsdag inklusiv MR og døgnblodtryk (x4), c) måltidstest (x4) og d) fedtbiopsi (x4). I alt giver det en ulempegodtgørelse på 10.000 kroner ved gennemførelse af det omfattende undersøgelsesprogram. Deltagelse i forsøget vil være et stort indgreb i forsøgspersonernes dagligdag. Interventionskosten skal indtages som en del af alle dagens hovedmåltider i 2 x 4 uger, hvilket for de fleste vil kræve en del planlægning. Samtidig vil personer med manglende opbevaringsmuligheder være nødt til at afhente interventionskost flere gange i løbet af fire uger. Kost- og afføringsregistreringen, som skal foretages over 3 dage, 4 gange i løbet af forsøget, vil ligeledes kræve en dedikeret indsats fra deltagerne. Selve undersøgelsesprogrammet omfatter som tidligere beskrevet bl.a. kikkertundersøgelser af tarmen, fedtvævsbiopsier, døgnblodtryk og blodprøver som skal gentages flere gange for hver deltager. Undersøgelserne indebærer ovenfor beskrevne risici og bivirkninger. Hver forsøgsdeltager skal således som minimum møde til 13 forsøgsdage, forudsat at alt forløber planmæssigt. Ved alle forsøgsdage skal deltagerne møde fastende, og forud for måltidstesten skal de desuden afholde sig fra alkohol og tobak. Efter fedtbiopsierne skal deltagerne undgå hård fysisk aktivitet i et par dage og afholde sig fra at gå i svømmehal indtil såret er helet. Der ydes desuden godtgørelse for kørselsudgifter og tabt arbejdsfortjeneste efter statens takster. Der gøres opmærksom på, at en del af forsøgsdagene foregår i weekenden, hvor der ikke er tabt arbejdsfortjeneste. Inklusionskriterierne bevirker, at den forventede gennemsnitlige alder for forsøgsdeltagerne ligger omkring 50-60 år. På denne baggrund findes det rimeligt, at der ydes ovennævnte ulempegodtgørelse. Der er ikke medicinalfirmaer involveret. Ingen i forskergruppen har privatøkonomiske interesser i projektet.***

# 12.0 Forskningsbiobank

## 12.1 Analyser i det konkrete projekt

***Indsamlet materiale til analyse i det konkrete projekt opbevares i etableret forskningsbiobank under Medicinsk Afdeling V og Medicinsk Endokrinologisk Afdeling (MEA), AUH.*** Prøvematerialerne opbevares aflåst i fryser ved -80^o^ C. Prøverne er kodet med deltagernes forsøgs-ID og kan kun sammenkædes med de enkelte deltagere ved hjælp af en ID-liste, som opbevares dobbeltaflåst på Medicinsk Afdeling V og MEA. ***Hovedparten af analyserne foretages i AUHs regi. Enkelte analyser af indsamlet materiale foretages i udlandet (USA), hvilket forsøgsdeltagerne oplyses om i den skriftlige deltagerinformation. Der sendes alene materiale med henblik på specifikke analyser, og restmateriale fra analyser foretaget uden for Medicinsk Afdeling V eller Medicinsk Endokrinologisk Afdeling destrueres. Der laves analyser følgende steder:***

1. ***Medicinsk Endokrinologisk Afdeling, AUH:***
   1. Fedtvævsprøver: 5 g per fedtbiopsi
2. ***V-Forskningslaboratorium, Medicinsk Afdeling V, AUH***
   1. ***Fæces: 3 x 5-10 g fæces som ”back-up” mhp yderligere mikrobiel karakterisering.***
   2. Fuldblod: Perifere blodmononukleære celler, plasma, serum analyseres og nedfryses ved -140°C og -80°C.
3. Institut for Husdyrbiologi og -sundhed, Aarhus Universitet (bilag 11):
   1. Urin: 2 x 3 ml til metabolomics per måltidstest.
   2. Fæcesprøver: 2-3 gram x 2 per endoskopi til SCFA- og proteinnedbrydningsanalyse.
   3. Plasma: 48 ml til metabolomics, SCFA og I-FABP
   4. Tarmbiopsier ved endoskopi:
      1. 20 mg til qPCR gemmes i 1.0 ml RNAlater. Biopsierne henstår ved stuetemperatur i 24 timer, hvorefter de opbevares ved -80° C.
      2. 20 mg til enzymaktivitet indfryses straks i flydende kvælstof og opbevares ved -80° C.
4. Maria Marco, UC Davis, USA :
   1. ***Fæcesprøve: 5-10 g til analyse mhp udvalgte bakteriegrupper (luminal mikrobiom)***

## 12.2 Materiale i forskningsbiobank til fremtidig forskning

***I forbindelse med projektet vil vi ved indhentning af skriftligt samtykke (bilag 5) bede om tilladelse til at opbevare resterende prøvemateriale efter endt analyse i det konkrete projekt i etablerede forskningsbiobanker ved dels Medicinsk Afdeling V, dels ved MEA, AUH, under den offentlige forvaltning og anmeldt til Datatilsynet via Region Midtjylland. Der opbevares ikke materiale uden for AUH. Brug af materiale fra forskningsbiobanken ved fremtidig forskning inden for forskningsfeltet vil kun finde sted efter forudgående konkret godkendelse fra Videnskabsetisk Komité.***

# 13.0 Behandling af personfølsomme data

Alle oplysninger om forsøgspersoner behandles efter Lov om behandling af personoplysninger og Sundhedsloven. Alle prøver vil blive kodet, så kun de forsøgsansvarlige kan forbinde prøverne til den enkelte forsøgsdeltager. Forsøgsresultater er fortrolige og udleveres ikke til udenforstående personer. Studiet bliver anmeldt til Datatilsynet og gennemføres i overensstemmelse med Helsinki-deklarationen.

# 14.0 Videnskabsetisk redegørelse

Forsøgene vil være med til at belyse om tilsætning af resistent stivelse og arabinoxylaner til en normal vestlig kost kan være med til at forebygge udvikling af type 2 diabetes, hjertekarsygdom og sygdom i tyktarmen. På baggrund af den stigende forekomst af disse sygdomme er det af samfundsøkonomisk interesse at finde non-farmakologiske tilgange til forebyggelse, herunder identificering af funktionelle fødevareingredienser.

De eksperimentelt fremstillede fødevareingredienser vil blive produceret ved hjælp af planter, der er godkendt til human ernæring. Produkterne er fremstillet under rene forhold og opfylder EFSA-standarderne.

Fordele for den enkelte forsøgsdeltager kan være at viden om egen helbredstilstand gennem klinisk og biokemisk undersøgelse i forbindelse med forsøgene. Endvidere udleveres en del af den daglige kost til 8 ugers ernæring.

Der ydes økonomisk kompensation for gener og ulemper i forbindelse med forsøget, som er beskrevet i økonomiafsnittet ovenfor.

Samlet vurderer vi, at værdien af den biomedicinske viden, som forsøgene kan bibringe, opvejer de gener og risici, som forsøgspersonerne udsættes for i forbindelse med forsøget.

# 15.0 Referencer

(1) Heidemann C, Schulze MB, Franco OH, et al. Dietary patterns and risk of mortality from cardiovascular disease, cancer, and all causes in a prospective cohort of women. Circulation 2008; 118: 230-7.

(2) Emberson JR, Whincup PH, Morris RW, et al. Lifestyle and cardiovascular disease in middle-aged British men: the effect of adjusting for within-person variation. Eur Heart J 2005; 26: 1774-82.

(3) Cornier MA, Dabelea D, Hernandez TL, et al. The metabolic syndrome. Endocr Rev 2008; 29: 777-822.

(4) Alberti KG, Eckel RH, Grundy SM, et al. Harmonizing the metabolic syndrome: a joint interim statement of the International Diabetes Federation Task Force on Epidemiology and Prevention; National Heart, Lung, and Blood Institute; American Heart Association; World Heart Federation; International Atherosclerosis Society; and International Association for the Study of Obesity. Circulation 2009; 120: 1640-5.

(5) Roberfroid M, Gibson GR, Hoyles L, et al. Prebiotic effects: metabolic and health benefits. Br J Nutr 2010; 104 Suppl 2: S1-63.

(6) Gemen R, De Vries PJF, Slavin JL. Relationship between molecular structure of cereal dietary fiber and health effects: Focus on glucose/insulin response and gut health. Nutr Rev 2011; 69: 22-33.

(7) Bach Knudsen KE, Serena A, Canibe N, et al. New insight into butyrate metabolism. Proc Nutr Soc 2003; 62: 81-6.

(8) McOrist AL, Miller RB, Bird AR, et al. Fecal butyrate levels vary widely among individuals but are usually increased by a diet high in resistant starch. J Nutr 2011; 141: 883-9.

(9) Denise Robertson M. Metabolic cross talk between the colon and the periphery: implications for insulin sensitivity. Proc Nutr Soc 2007; 66: 351-61.

(10) Hamer HM, Jonkers D, Venema K, et al. Review article: the role of butyrate on colonic function. Aliment Pharmacol Ther 2008; 27: 104-19.

(11) Canani RB, Costanzo MD, Leone L, et al. Potential beneficial effects of butyrate in intestinal and extraintestinal diseases. World J Gastroenterol 2011; 17: 1519-28.

(12) Gao Z, Yin J, Zhang J, et al. Butyrate improves insulin sensitivity and increases energy expenditure in mice. Diabetes 2009; 58: 1509-17.

(13) Robertson MD, Currie JM, Morgan LM, et al. Prior short-term consumption of resistant starch enhances postprandial insulin sensitivity in healthy subjects. Diabetologia 2003; 46: 659-65.

(14) Johnston KL, Thomas EL, Bell JD, et al. Resistant starch improves insulin sensitivity in metabolic syndrome. Diabet Med 2010; 27: 391-7.

(15) Lu ZX, Walker KZ, Muir JG, et al. Arabinoxylan fibre improves metabolic control in people with type II diabetes. Eur J Clin Nutr 2004; 58: 621-8.

(16) Garcia AL, Otto B, Reich SC, et al. Arabinoxylan consumption decreases postprandial serum glucose, serum insulin and plasma total ghrelin response in subjects with impaired glucose tolerance. Eur J Clin Nutr 2007; 61: 334-41.

(17) Kolovou GD, Mikhailidis DP, Kovar J, et al. Assessment and clinical relevance of non-fasting and postprandial triglycerides: an expert panel statement. Curr Vasc Pharmacol 2011; 9: 258-70.

(18) Carstensen M, Thomsen C, Gotzsche O, et al. Differential postprandial lipoprotein responses in type 2 diabetic men with and without clinical evidence of a former myocardial infarction. Rev Diabet Stud 2004; 1: 175-84.

(19) Dongowski G, Huth M, Gebhardt E. Steroids in the intestinal tract of rats are affected by dietary-fibre-rich barley-based diets. Br J Nutr 2003; 90: 895-906.

(20) Thomsen C, Rasmussen O, Lousen T, et al. Differential effects of saturated and monounsaturated fatty acids on postprandial lipemia and incretin responses in healthy subjects. Am J Clin Nutr 1999; 69: 1135-43.

(21) Thomsen C, Storm H, Holst JJ, et al. Differential effects of saturated and monounsaturated fats on postprandial lipemia and glucagon-like peptide 1 responses in patients with type 2 diabetes. Am J Clin Nutr 2003; 77: 605-11.

(22) Nordgaard I, Hove H, Clausen MR, et al. Colonic production of butyrate in patients with previous colonic cancer during long-term treatment with dietary fibre (Plantago ovata seeds). Scand J Gastroenterol 1996; 31: 1011-20.

(23) Beards E, Tuohy K, Gibson G. A human volunteer study to assess the impact of confectionery sweeteners on the gut microbiota composition. Br J Nutr 2010; 104: 701-8.

(24) Matsuda M, DeFronzo RA. Insulin sensitivity indices obtained from oral glucose tolerance testing: comparison with the euglycemic insulin clamp. Diabetes Care 1999; 22: 1462-70.

(25) Matthews DR, Hosker JP, Rudenski AS, et al. Homeostasis model assessment: insulin resistance and beta-cell function from fasting plasma glucose and insulin concentrations in man. Diabetologia 1985; 28: 412-9.

(26) Rico-Sanz J, Thomas EL, Jenkinson G, et al. Diversity in levels of intracellular total creatine and triglycerides in human skeletal muscles observed by (1)H-MRS. J Appl Physiol 1999; 87: 2068-72.

(27) Thomas EL, Hamilton G, Patel N, et al. Hepatic triglyceride content and its relation to body adiposity: a magnetic resonance imaging and proton magnetic resonance spectroscopy study. Gut 2005; 54: 122-7.

(28) Francis CY, Morris J, Whorwell PJ. The irritable bowel severity scoring system: a simple method of monitoring irritable bowel syndrome and its progress. Aliment Pharmacol Ther 1997; 11: 395-402.

(29) Svedlund J, Sjodin I, Dotevall G. GSRS--a clinical rating scale for gastrointestinal symptoms in patients with irritable bowel syndrome and peptic ulcer disease. Dig Dis Sci 1988; 33: 129-34.

(30) Lewis SJ, Heaton KW. Stool form scale as a useful guide to intestinal transit time. Scand J Gastroenterol 1997; 32: 920-4.

(31) Stjernman H, Granno C, Jarnerot G, et al. Short health scale: a valid, reliable, and responsive instrument for subjective health assessment in Crohn's disease. Inflamm Bowel Dis 2008; 14: 47-52.

(32) Hjortswang H, Jarnerot G, Curman B, et al. The Short Health Scale: a valid measure of subjective health in ulcerative colitis. Scand J Gastroenterol 2006; 41: 1196-203.

(33) Hvas CL, Kelsen J, Agnholt J, et al. Crohn's disease intestinal CD4+ T cells have impaired interleukin-10 production which is not restored by probiotic bacteria. Scand J Gastroenterol 2007; 42: 592-601.

(34) Agnholt J, Kaltoft K. In situ activated intestinal T cells expanded in vitro--without addition of antigen--produce IFN-gamma and IL-10 and preserve their function during growth. Exp Clin Immunogenet 2001; 18: 213-25.

(35) Bertram HC, Bach Knudsen KE, Serena A, et al. NMR-based metabonomic studies reveal changes in the biochemical profile of plasma and urine from pigs fed high-fibre rye bread. Br J Nutr 2006; 95: 955-62.

(36) Rabeneck L, Paszat LF, Hilsden RJ, et al. Bleeding and perforation after outpatient colonoscopy and their risk factors in usual clinical practice. Gastroenterology 2008; 135: 1899,1906, 1906.e1.

Bilag:

1. Lægmandsresumé
2. Deltagerinformation
3. Forsøgspersoners rettigheder ved deltagelse i et biomedicinsk forsøg
4. Screeningsspørgeskema før inklusion
5. Samtykkeerklæring
6. Annonce til rekruttering af forsøgsdeltagere
7. Kostdagbog
8. Tarmdagbog
9. Volumenberegning for blodprøvetagning
10. Metalskema
11. Biobank
12. Dokumentation for forsøgsansvarliges identitet
13. Forsøgsansvarliges Curriculum Vitae
