# Supplementary material for: Effects of Arabinoxylan and Resistant Starch on Intestinal Microbiota and Short-Chain Fatty Acids in Subjects with Metabolic Syndrome: A Randomised Crossover Study
Source: PLoS One. 2016 Jul 19;11(7):e0159223. doi: 10.1371/journal.pone.0159223 (PMC4951149; doi:10.1371/journal.pone.0159223)
Supplement: S1 Table — (DOCX) [file pone.0159223.s003.docx]

**S1 Table:** **Nutritional composition of the key foods on a dry matter (DM) basis for the healthy-carbohydrate diet (HCD) and Western-style diet (WSD).**

|  | **HCD** | | | | | | | **WSD** | | | | | |
| --- | --- | --- | --- | --- | --- | --- | --- | --- | --- | --- | --- | --- | --- |
| Key foods  (%) | **Rye flakes** | **Rye bread** | **Combo rolls** | **Rye pasta** | **Combo pancake** | **Potato starch** | **Smoo-thie** | **Corn-flakes** | **Toast bread** | **Spelt rolls** | **Wheat pasta** | **Wheat pancake** | **Smoo-thie** |
| DM % | 91.8 | 56.9 | 62.0 | 90.5 | 46.2 | 88.9 | 14.9 | 98.3 | 63.7 | 66.0 | 90.5 | 37.0 | 14.9 |
| Ash | 1.5 | 3.5 | 2.3 | 0.9 | 3.3 | 0.3 | 2.6 | 1.4 | 2.6 | 2.3 | 0.9 | 3.3 | 2.6 |
| Protein | 10.3 | 8.5 | 14.6 | 10.3 | 15.8 | 0.5 | 2.7 | 7.5 | 13.5 | 16.0 | 14.9 | 18.5 | 2.7 |
| Fat | 1.7 | 2.3 | 2.2 | 1.7 | 25.7 | 0.0 | 0.2 | 0.7 | 3.3 | 3.2 | 2.0 | 25.9 | 0.2 |
| DCs | 67.8 | 61.2 | 60.0 | 72.0 | 30.0 | 37.1 | 68.4 | 86.0 | 76.4 | 74.4 | 79.1 | 48.4 | 68.4 |
| Sugars^a^ | 2.4 | 3.0 | 2.2 | 2.5 | 1.7 | 0.1 | 66.1 | 8.1 | 2.3 | 1.5 | 1.1 | 0.6 | 66.1 |
| Lactose | 0.0 | 0.0 | 0.0 | 0.0 | 4.7 | 0.0 | 0.0 | 0.0 | 0.0 | 0.0 | 0.0 | 6.0 | 0.0 |
| DS | 65.5 | 58.3 | 57.8 | 69.5 | 23.7 | 37.0 | 2.3 | 77.9 | 74.1 | 72.9 | 78.0 | 41.8 | 2.3 |
| NDCs | 17.5 | 18.3 | 15.8 | 12.4 | 18.5 | 61.0 | 8.4 | 3.6 | 5.3 | 3.9 | 5.1 | 3.3 | 8.4 |
| RS_enz_^b^ (RS_dmso_^c^) | 0.4(0.2) | 2.1(1.3) | 5.4(4.2) | 1.1(0.7) | 6.5(5.5) | 59.6(0) | 0(0) | 1.8(0.8) | 1.2(0.7) | 0.6(0.4) | 0.9(0.5) | 0.7(0.1) | 0(0) |
| NSPs | 14.1 | 14.4 | 9.7 | 9.7 | 11.0 | 1.1 | 3.3 | 1.7 | 3.4 | 2.8 | 3.3 | 2.1 | 3.3 |
| Cellulose | 1.0 | 1.6 | 1.9 | 0.9 | 2.1 | 0.0 | 0.9 | 0.4 | 0.5 | 0.3 | 0.3 | 0.2 | 0.9 |
| AX | 9.1 | 8.4 | 3.4 | 5.6 | 3.3 | 0.0 | 0.4 | 0.3 | 1.7 | 1.3 | 1.8 | 0.9 | 0.4 |
| A:X ratio | 0.67 | 0.66 | 0.79 | 0.76 | 0.82 | - | 1.68 | 1.08 | 0.86 | 0.96 | 0.90 | 0.95 | 1.68 |
| LMW NDCs | 3.0 | 1.8 | 0.8 | 1.6 | 1.1 | 0.3 | 5.1 | 0.1 | 0.6 | 0.4 | 0.9 | 0.5 | 5.1 |
| Fructan | 3.0 | 1.8 | 0.4 | 1.6 | 0.3 | 0.0 | 5.0 | 0.0 | 0.3 | 0.1 | 0.5 | 0.3 | 5.0 |
| AXOS | 0.0 | 0.0 | 0.4 | 0.0 | 0.7 | 0.3 | 0.2 | 0.1 | 0.3 | 0.3 | 0.4 | 0.2 | 0.2 |
| Lignin | 1.7 | 2.0 | 1.4 | 1.4 | 1.8 | 0.4 | 1.9 | 1.1 | 0.9 | 1.1 | 0.7 | 1.2 | 1.9 |
| Total DF^d^ | 15.8 | 16.4 | 11.1 | 11.1 | 12.8 | 1.5 | 10.3 | 4.7 | 6.2 | 5.0 | 5.8 | 4.5 | 10.3 |

A:X ratio, arabinose to xylose ratio; AX, arabinoxylan; AXOS, arabinoxylan oligosaccharides; DCs, digestible carbohydrates; DF, dietary fibre; DS, digestible starch; LMW, low molecular weight; NDCs, non-digestible carbohydrates; NSPs, non-starch polysaccharides; RS, resistant starch. ^a^Calculated as the sum of glucose, fructose and sucrose. ^b^RS_enz_ was determined by Megazyme assay (Megazyme International). ^c^RS_DMSO_ was determined as described by Bach Knudsen ^(35)^. ^d^Calculated as the sum of non-digestible carbohydrates and lignin.
