# Supplementary material for: Effects of Arabinoxylan and Resistant Starch on Intestinal Microbiota and Short-Chain Fatty Acids in Subjects with Metabolic Syndrome: A Randomised Crossover Study
Source: PLoS One. 2016 Jul 19;11(7):e0159223. doi: 10.1371/journal.pone.0159223 (PMC4951149; doi:10.1371/journal.pone.0159223)
Supplement: S2 Table — Values are presented as medians and interquartile ranges in brackets. (DOCX) [file pone.0159223.s004.docx]

**S2 Table: Bodyweight, body fat percentage and waist circumference at run-in and at the end of western-style diet (WSD) and healthy-carbohydrate diet (HCD).** Values are presented as medians and interquartile ranges in parentheses.

|  | **WSD** | | **HCD** | |  |  |  |  |
| --- | --- | --- | --- | --- | --- | --- | --- | --- |
|  | Run-in | End | Run-in | End | *P-*value  WSD-Run-in vs  HCD-Run-in | *P*-value  WSD End vs HCD End | *P*-value  ΔWSD vs  ΔHCD ^a^ | *P*-value  Total study time ^b^ |
| Body weight (kg) | 97.6  (88.4-109.9) | 98.0  (89.6-111.2) | 97.6  (89.3-114.0) | 97.7  (88.7-112.6) | 0.27 | 0.65 | 0.97 | 0.15 |
| Body fat percentage (%) | 33.3  (30.1-39) | 33.3  (29-39.9) | 33.1  (29.9-39) | 33.2  (29.9-38.6) | 0.38 | 0.92 | 0.38 | 0.54 |
| Waist circum-ference (cm) | 108  (104-119) | 109  (104-119) | 112.5  (103-119) | 109  (104-119) | 0.34 | 1 | 0.30 | 0.24 |

^a^ ΔWSD = WSD end – WSD run-in. ΔHCD = HCD end – HCD run-in. ^b^Total study time = value at the end of the study compared to the first run-in value

(Paired t-test)
